# Relaxation of the Adsorbed Material and Shadowing Effects on the Shape and Size of Electrodeposited Dendrites

**DOI:** 10.1021/acs.langmuir.5c00740

**Published:** 2025-04-25

**Authors:** Dung di Caprio, Abdelhafed Taleb, Fábio D. A. Aarão Reis

**Affiliations:** † Chimie ParisTech - CNRS, Institut de Recherche de Chimie Paris, PSL Research University, 75005 Paris, France; ‡ Instituto de Física, Universidade Federal Fluminense, Avenida Litorânea s/n, 24210-340 Niterói, RJ, Brazil; ¶ Sorbonne Universités, 75231 Paris, France; # ITODYS, CNRS UMR-7086, Paris cité Universite, 75013 Paris, France

## Abstract

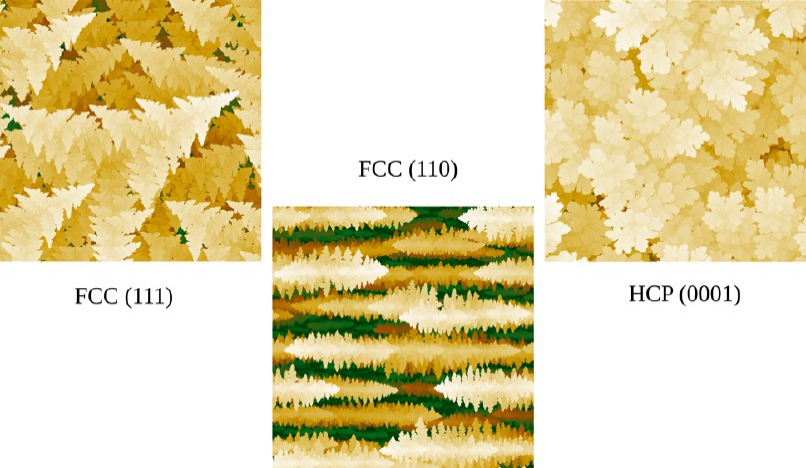

Materials with dendritic morphologies exhibit large surface areas that improve the catalytic, optical, and wetting properties but have ambiguous effects in batteries, so modeling their growth may help find the best operation conditions in each case. Kinetic Monte Carlo simulations are used here to study a metal electrodeposition model that represents the interplay between diffusive cation flux in the electrolyte and surface diffusion of adsorbed atoms (adatoms) with electrodes perpendicular to the gradient of the electrolyte concentration and different crystallographic orientations. In FCC lattices, dendrites with a pine tree shape are formed for all orientations, with dominant (111) surfaces and with trunks propagating in [001] and equivalent directions. However, with (110) and (111) substrates, secondary branches do not grow because the inclined primary branches block the cation flux (shadowing effect), so the dendrites may have a leaf-like shape. Some morphologies obtained here resemble those of the silver and gold electrodeposits. The extension to electrodeposition of HCP crystals with (0001) substrates shows the formation of leaf-like dendrites with a hexagonal symmetry. In both lattices, hierarchically organized structures appear for model parameters that warrant large diffusion lengths of adsorbed atoms on flat planes (typically coordination numbers *n* ≤ 4) and their stability at low-energy configurations (*n* ≥ 7). Average dendrite widths scale approximately with the diffusion length from adsorption to permanent incorporation to the crystal. These results show that dendrite widths are directly related to the relaxation of the electrodeposited material, and their crystallography is controlled by the energetics of the relaxation, but their visual appearance may depend on their angles with the electrode. In the range of model parameters where the coordination number weakly affects the diffusion of adsorbed atoms, the dendrites become rounded and have flower-like shapes. Possible effects of the orientation on the physicochemical properties of thin dendritic films are discussed.

## Introduction

Dendrites are hierarchically organized structures at nano- or micro-scales whose open porosity and high specific surface area are beneficial for catalytic reactions,^1−5^ technologies based on surface-enhanced Raman spectroscopy,^6−10^ superhydrophobic coatings,^11−14^ and photovoltaic devices,^15^ among other applications. Conversely, the growth of lithium dendrites in batteries is deleterious because it may lead to short circuits between the electrodes.^16−18^ The dendrite morphology plays an important role in each application, which justifies the improvement of material production techniques. Among these techniques, electrochemical deposition appears as a low-cost option with a high ability to tune the morphology of metallic films and nanoparticles.^1,4−7,9,19−21^ Compact and porous films, faceted microparticles, ramified structures with the geometry of random fractals, and hierarchical structures of several materials were formed with this technique. External parameters, such as the applied potential (or applied current), temperature, pH, and ion concentrations in solution, are responsible for controlling the microscopic processes that produce these shapes.

If the deposition is limited by diffusion in the electrolyte and the relaxation of the adsorbed material is negligible, then highly branched structures with no organization are produced. They can be observed in the electrodeposition of some metals^22^ and may be described by the well-known model of diffusion-limited aggregation (DLA).^23,24^ However, several studies recognize the importance of diffusion of the adsorbed material in shaping the electrodeposited dendrites.^5,25−37^ For instance, in a transmission electron microscopy (TEM) study of electrodeposited silver, the dendrites had crystalline cores surrounded by amorphous layers with ∼5 nm thickness, and the crystallization proceeded by relaxation of these layers.^25^ Moreover, a TEM study of electrodeposition of lead dendrites showed that they grew at the expense of the relaxation of a surrounding polycrystalline phase.^38^ Thus, the formation of low-energy (organized) configurations is expected on short length scales.

Models for the formation of thin films, nanoparticles, and other nanostructures are important because their morphologies are related to the microscopic dynamics and may predict routes to improve their physical and chemical properties. In the light of the above-mentioned TEM studies, a recent metal electrodeposition model assumed that adsorbed atoms (adatoms) execute surface diffusion only during a short time scale after adsorption,^39^ which is a mechanism termed limited mobility. The interplay among this relaxation mechanism, the diffusive cation flux in the electrolyte, and rapid electrochemical reactions allows the organization and stability of low-energy configurations at the nano- or micro-scales, while shadowing by surface protuberances control the morphologies at larger scales. Indeed, kinetic Monte Carlo (KMC) simulations of this model showed the nucleation and growth of hierarchically organized structures^39,40^ and described the morphology evolutions of electrodeposited silver^40^ and iron.^41^ However, other electrodeposition models have assumed collective adatom diffusion with thermally activated rates (similar to vapor deposition models^42−44^) and showed formation of DLAs, compact films, and columnar deposits,^45−51^ but they do not show the formation of organized dendritic patterns.

The present work extends the previous study of the limited mobility electrodeposition model to different crystallographic orientations of the electrodes relative to the direction of the concentration gradient in the electrolyte. Solids with an FCC and HCP structure are considered. In different FCC substrates, KMC simulations show that the dendrites propagate in the same crystallographic directions and have the same dominant (111) surfaces, but visually different dendrite shapes may appear due to shadowing of the growth of some secondary and tertiary branches. The dendrites in HCP lattices are organized according to the hexagonal symmetry, whereas flower-like dendrites are obtained in narrow ranges of parameters in both lattices. This variety of dendrite shapes resemble those of some studies with gold, silver, and Ag–Cu alloys.^1,52−54^ Despite these differences, in all lattices and orientations, the measured dendrite widths are shown to be on the same order of magnitude as the adatom diffusion lengths from adsorption to crystallization. The results show how the energetics of adatom relaxation and the cation flux control the dendrite shapes at different length and time scales, which may help change these shapes by tuning the temperature, applied current, or other parameters that affect the surface relaxation.

## Materials and Methods

### Electrodeposition Model

The model is defined in a Bravais lattice, which may be simple cubic (SC), FCC, or ideal HCP. The electrode is planar with the normal vector denoted as , and nucleation is allowed in all electrode sites (representing a high density of nucleation sites). All sites above that plane (i.e., in the direction of ) initially contain a supported electrolyte, and the deposit will grow in this region. The size of the simulation cell in the direction perpendicular to  (i.e., the lateral size) is denoted as *L*, and periodic boundaries are adopted in these directions to avoid size effects.

**Figure 1:**
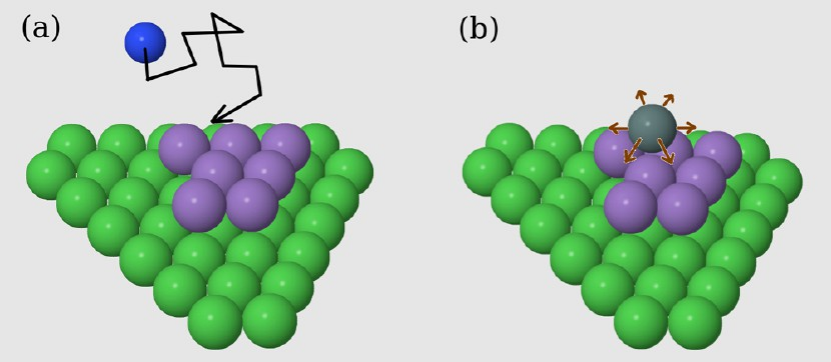
Scheme of the electrodeposition model in HCP lattices. (a) Random walk of a cation (blue sphere) in the electrolyte until it reaches a position where it has 3 NNs of the deposit (magenta spheres). Green spheres are the substrate sites. (b) A mobile atom (gray sphere) is formed by the electrochemical reaction and the directions to which the first hop can be accepted are shown (brown arrows). Observe that the hop directions have the same directions of the substrate bonds in this lattice.

Figure 1a illustrates the diffusion of a cation in the electrolyte and the growing deposit in the HCP lattice with the electrode in the basal plane (0001). Similar illustrations with SC (001) and FCC (001) electrodes are shown in previous publications.^39,40^ Each cation is released well above the deposit in order to simulate the effect of a depleted layer near the electrode. The cation executes a random walk that ends when it reaches a site with at least one occupied nearest neighbor (NN), which may be a site of the electrode or a previously deposited atom. The cation is instantaneously reduced at this final position, and a mobile atom is formed there.

The adsorbate relaxation is represented by a surface random walk of the mobile atom with quenched positions of the previously deposited atoms (i.e., a stable crystal configuration). Each atom attempts to execute *G* hops to NN sites and permanently aggregates at the final position after these attempts. In each hop attempt, a target site is randomly chosen among the 12 NNs of the current position. If the target site is empty and if it has at least one occupied NN, the hop is executed with probability
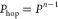
1where *P* < 1 and *n* is the number of occupied NNs at the current position (including atoms of the aggregate and electrode sites). If any of these two conditions fails, the hop is not executed, and the mobile atom remains at its current position. In both cases, the number of available hop attempts decreases by one unit. Figure 1b illustrates the possible hops of a mobile atom in the HCP lattice. In the SC lattice, the only difference in these rules is that the target site is chosen among NN and next NN (NNN) sites, which allows a mobile atom to contour sharp edges.^39,55^ The condition that the target site of a hop has at least one occupied NN implies that there is no desorption.

### Physical Interpretation of the Model

Defects in electrode surfaces are generally expected to act as preferential points for cluster nucleation. However, with suitable methods for substrate modification, it is possible to obtain almost uniform nucleation at the nanoscale. This is the case of the patterning of highly oriented pyrolitic graphite (HOPG) substrates by Au nanoparticles with average diameters of 4 nm.^56,57^ Recently, thin coatings and nanoparticle patterns emerged as tools to obtain more uniform deposition and avoid dendrite formation on zinc anodes of aqueous zinc ion batteries.^58−61^ Thus, initially planar electrodes with nucleation at all lattice sites were a realistic condition for our study, although the role of dendrite growth differs among these applications.

The model also assumes that the reduction of the cation in contact with the electrode or the deposit is instantaneous. This implies that the electrochemical reaction is much faster than the cation transport in the electrolyte and much faster than the diffusion of the adsorbed atoms (mobile atoms). The reactions in contact with the electrode and with the deposit represent nucleation and growth processes.

Equation 1 for the hop probability implies that the atom mobility decreases as the coordination number *n* increases, which corresponds to an increase in the bond energy. If the mobile atom is in a region where most empty sites have *n* NNs, then the number of hops is on the order of *GP*_hop_ = *GP*^*n*–1^. On average, this atom rapidly moves at points where *GP*^*n*–1^ ≫ 1, moves only in a close neighborhood of points where *GP*^*n*–1^ ∼ 1, and is effectively frozen at points where *GP*^*n*–1^ ≪ 1. A comparison with models of thermally activated diffusion suggests an Arrhenius form^55,62^
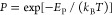
2so that *P* increases with the temperature. In this context, eq 1 implies that the activation energy for the hops of mobile atoms is additive over the NNs, with no dependence on the orientation of these NNs.

The number of hop attempts, *G*, is proportional to the average time that a deposited atom relaxes before crystallization. This time depends on the properties of the surface layers where the cations are reduced, which may be controlled by several parameters such as the applied current, the pH, the ion concentration in solution, and the temperature. If the temperature increases with the other parameters kept constant, the mobility in these surface layers is expected to increase, so the form *G* = *G*_0_ exp[−*E*_G_/(*k*_B_*T*)] is also justified.^62^ However, the estimation of the amplitude *G*_0_ and activation energies *E*_P_ and *E*_G_ for a given process depends on controlled studies at different temperatures and different currents, which are seldom available.

The choice of the main parameters *G* and *P* depends on computational limitations and on the interest in the achieved morphologies. Computer time and memory usually restrict simulations to *G* < 10^5^ in cells with ≳10^3^ lattice units in each spatial direction. Even for the largest values of *G*, *P* ≤ 0.01 generally produces randomly branched deposits with a DLA shape,^39,40,62^ which have been studied since a long time.^24^ However, for *G* ≲ 10^5^, hierarchically organized structures (dendrites) are obtained for *P* ≥ 0.02, so this work and other recent studies have focused on this parameter range. The size of the simulated structures may be much smaller than those of experiments, so extrapolations to larger *G* values may be necessary to find the suitable modeling parameters; for instance, for modeling silver electrodeposits on substrates templated with gold nanoparticles, a recent study suggested *G* ∼ 10^11^ and *P* ∼ 0.03.^40^

### Simulation Details

The novel KMC simulations presented in this work were performed in FCC lattices with planar substrates (111) and (110) and in HCP lattices with the (0001) substrate. Some results of previous simulations in SC and FCC lattices, both with (001) substrates, are also presented for comparison.^39,40^ The lateral and vertical sizes of the simulation cells are *L* = 1536 in lattice units.

The cations are released at heights of 45 lattice units above the maximal height of the deposit, with the heights measured in the  direction defined by the substrate.

When a deposit grows by the sequential release of cations and subsequent diffusion of mobile atoms, the values of *G* and *P* remain constant. This means that quantities such as the applied current, the pH, the ion concentration in solution, and the temperature remain approximately constant during the growth. The values of *G* considered in the simulations ranged from 5 × 10^2^ to 5 × 10^4^ and the values of *P* ranged from 0.025 to 0.3. For most parameter sets (*G*, *P*), 10 deposits were grown, but for the calculation of average dendrite widths, 100 deposits were grown for selected values of these parameters.

The average width *W* of the dendrites is calculated for each parameter set by the same method as in a previous work,^40^ as follows. First, five cross sections perpendicular to  are chosen near 2/3 of the total height of the simulation cell, where the densities of all deposits reach an approximately height-independent value. In each cross-section, the *x* and *y* directions are defined. For each value of *y*, the lengths of segments of connected (NN) atoms along the *x* direction are measured, with periodic boundaries being considered. The dendrite width *W* is the average of these lengths over all values of *y*, over the five cross sections, and over the different deposits grown with each parameter set.

The simulations were run on NVIDIA Tesla K80 graphic cards in the CUDA environment for parallelization. In one simulation step, the number of atoms corresponding to an entire plane is processed, in our case 2.35 × 10^6^ atoms. The number of deposited planes corresponds to what we refer to later as the nominal thickness *T*. Accidentally, in this process, more than one mobile atom may reach the same final site as several atoms are processed simultaneously. When this occurs, the aggregation of these atoms is discarded. The number of discarded atoms is recorded, and at the end of the simulation step, additional atoms are released. In order to maintain a parallel calculation efficiency, only multiples of 512 particles are simultaneously released, which may not precisely match the number of missing atoms. Furthermore, during this sequence, supplementary collisions may occur. However, whichever the simulation parameters, we note that at the end of a step, the number of missing atoms remains far below 1% of the number of deposited atoms. Moreover, as we continuously track missing atoms and steadily compensate for them, over the entire simulation, the relative difference between the expected number of deposited atoms and the effective number is less than 10^–6^. Therefore, the algorithm to compensate collision events, adapted to parallel calculations, proves to be efficient. The simulations end when the cell height is insufficient to release new cations.

## Results and Discussion

### Nuclei Formation on Planar Electrodes

Here, we show that the model with planar substrates leads to an approximately uniform nucleation and how the choice of the lattice geometry controls the shape of the growing material. A part of this section is a review of previous studies.

**Figure 2:**
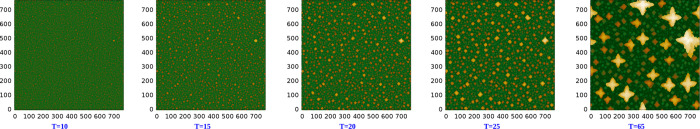
Images of deposits grown in FCC lattices with (001) substrate, *G* = 5 × 10^3^, and *P* = 0.2. In all panels, the sequence of colors with increasing heights is roughly dark green, light green, brown, tan, cream, and white. All lengths are in units of the lattice constant, and *T* is the nominal thickness.

Figure 2 shows images of the electrodeposition with parameters *G* = 5 × 10^3^ and *P* = 0.2, with the deposit constrained to an FCC lattice and with substrate orientation (001). The corresponding nominal thickness *T* is also shown. At *T* = 10, small fluctuations in the thickness are observed in the whole deposit, and a large bump on the right side of the image is visibly clear. Surface fluctuations are naturally formed due to the randomness of the flux and consequent randomness of aggregation, as explained by kinetic roughening theories.^63^ At *T* = 15 and *T* = 20, the bump on the right side has grown as a pyramid with (111) facets; similar pyramids are spread throughout the surface at points where the initial fluctuations were observed. Due to the diffusive cation flux, the surface hills become preferential points for cation reduction because they are more easily reached by these cations than the surface valleys (tip effect). At *T* = 25, a deformation of the largest pyramid is observed, with the stretching of branches in the (±1,0,0) and (0,±1,0) directions. At a longer time, *T* = 65, a distribution of dendrites with long branches in these directions is observed. The areal density of dendrites is much smaller than the density of initial nuclei due to the shadowing of the region near the tallest dendrites, which can be interpreted as unstable growth.

**Figure 3:**
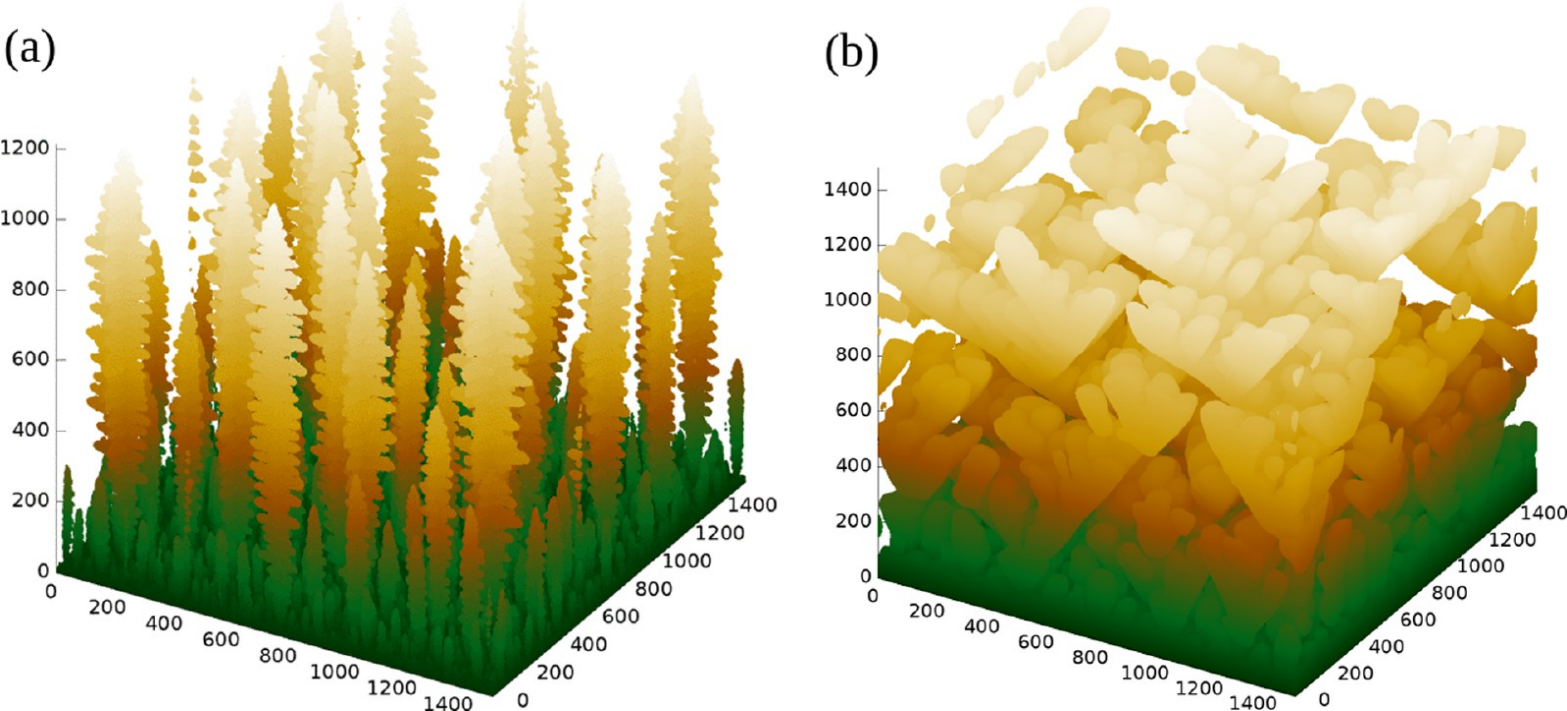
Perspective views of deposits that occupy most of the simulation cells, grown with *G* = 5 × 10^3^ and *P* = 0.2, on (a) FCC(001) and (b) SC(001) substrates. All lengths are expressed in units of the lattice constant.

At much longer times, the perspective view in Figure 3a shows that the dendrites have pine tree shapes, as formerly discussed in the context of silver electrodeposition.^40^ Inspection of the bottom layers of this image confirms that a large number of initial trees stopped growing due to the shadowing effect of the tallest trees. The trunks of these trees are aligned with the [001] direction (perpendicular to the substrate), the secondary branches are in the [±1,0,0] and [0,±1,0] directions, and tertiary branches are perpendicular to the secondary ones. The growth of tertiary branches in the [0,0,–1] direction is restricted due to the shadowing effect of the secondary branches. The surface of the pine trees is mostly of (111) orientation, as shown in a previous work.^40^

Figure 3b shows a perspective view of the growth in SC lattices with (001) substrate and the same parameters *G* and *P*. In this 3D view, the dendrites seem to have a flower-like shape, while a previous work showed that each branch has the shape of a maple leaf.^39^ This shows how a visual interpretation of the dendrite shape can be difficult. Anyway, in all cases, these dendrites are significantly different from those grown on the FCC(001) substrate, although the model parameters are the same. Another interesting difference is that a thin compact layer is formed on the SC(001) substrate (Figure 3b), which is correlated with the uniform nucleation.^55,62^ However, this compact layer is observed in FCC lattices only for much larger values of the parameter *G*.^40^

### Dendrite Morphology with FCC(111) Substrates

**Figure 4:**
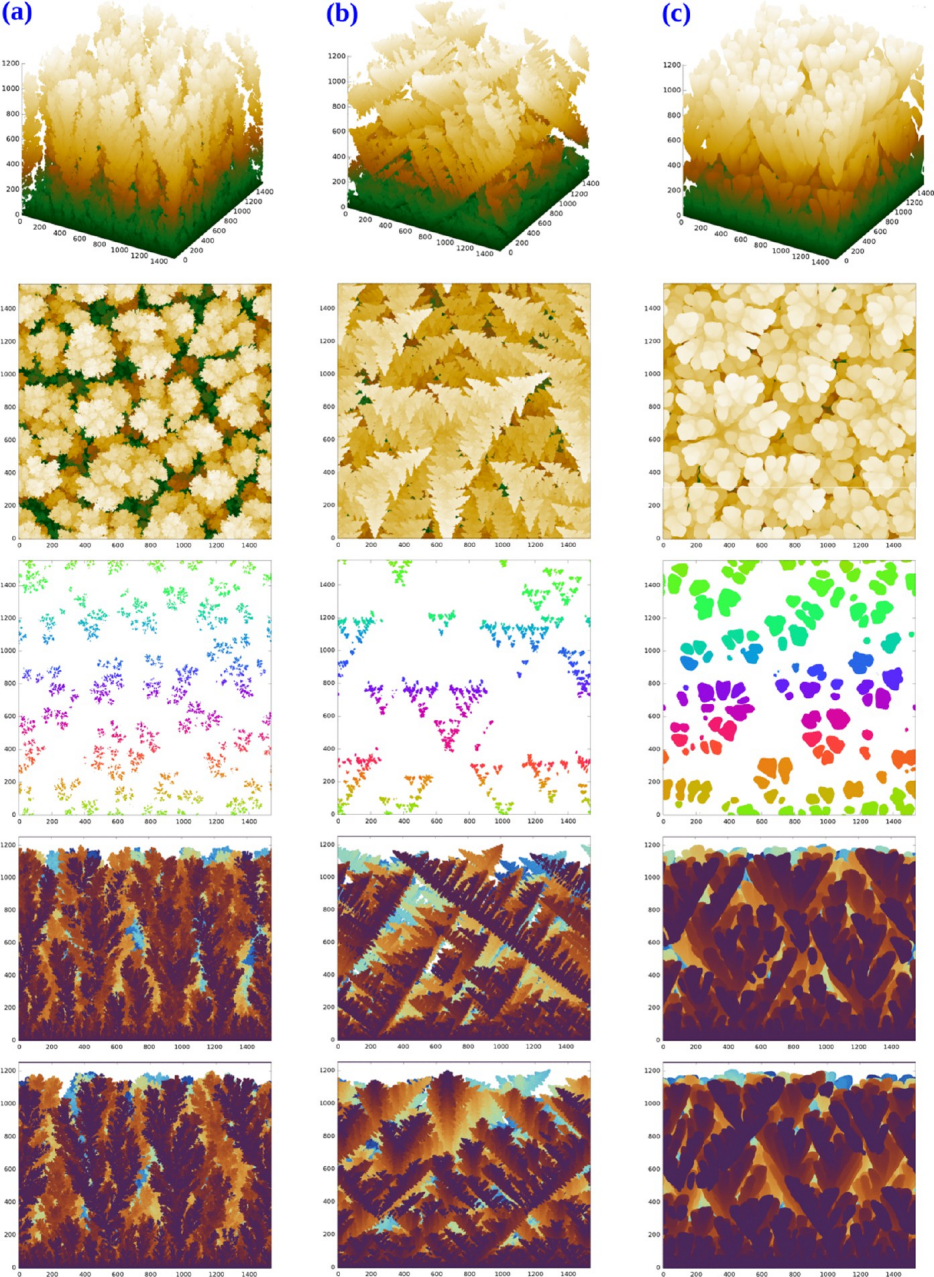
From top to bottom, perspective views, top views, and cross sections at ≈2/3 of the maximal height, and two lateral views of deposits grown on FCC(111) substrates with *G* = 10^4^ and (a) *P* = 0.025, (b) *P* = 0.1, and (c) *P* = 0.4. In the cross sections, the colors indicate connected clusters in 2D. All lengths are in units of the lattice constant.

Figure 4a–c shows views and cross sections of deposits grown with *G* = 10^4^ and respective values of *P* = 0.025, 0.1, and 0.4. These images illustrate three morphologies observed with this substrate orientation, which is the lowest-energy plane of the FCC lattice. Figures S1–S7 of the Supporting Information show images obtained with other parameter sets.

For *P* = 0.025 (Figure 4a), the deposit has disordered branches with no apparent organization at short or large length scales. Similar deposits are obtained in diffusion-limited deposition,^24^ which is the limit *G* = 0 of the model (note that the original DLA model considers a point seed^23^). The same result is obtained in all simulations with *P* = 0.025 and *G* ≤ 2 × 10^4^, as well as in all simulations with *G* = 5 × 10^2^ and all values of *P* (Figures S1–S7).

For *P* = 0.1 (Figure 4b), the deposit has organized structures that, when observed from the top, have a leaf-like shape. Similar patterns are obtained, for instance, with (*G* = 10^3^, 0.2 ≤ *P* ≤ 0.3), (*G* = 10^4^, 0.05 ≤ *P* ≤ 0.3), and (*G* = 2 × 10^4^, 0.025 ≤ *P* ≤ 0.3) (Figures S1–S7). These ranges of *G* and *P* imply large numbers of hops (*GP*^*n*–1^ ≳ 1) in the highest-energy configurations with *n* ≤ 4, which is the case of mobile atoms on the (111) and (001) plateaus. They also imply a small probability of hopping (*GP*^*n*–1^ ≪ 1) in the lowest-energy configurations with *n* ≥ 8, which belong to low-energy planes. These conditions ensure the stability of organized configurations at small length scales (characteristic of the dendrites) but with the necessary atom mobility on low-energy planes to reach these configurations.

The primary branches (trunk) of the dendrites are mostly oriented in the directions [100], [010], and [001], consistently with the angles of 120° between the trunks in the top views of Figure 4b; at this point, observe that the projections of these three vectors onto the (111) plane have the respective directions of [2,–1,–1], [−1,2,–1], and [−1,–1,2]. In the lateral views, some secondary branches have the same dark-brown color of their trunks, which indicates that they are at the same depth. Since the secondary branches are perpendicular to the trunks, these branches also propagate in directions [100], [010], and [001].

Thus, the structure of these dendrites has similarities to the pine trees obtained with the (001) substrate (Figure 3a). The difference is the absence of secondary and tertiary branches in the directions [−1,0,0], [0,–1,0], and [0,0,–1], which are shadowed because the incoming cation flux is in the [0,0,–1] direction. Thus, the leaf-like shape in the perspective and top views of Figure 4b results from the almost complete suppression of two neighboring secondary branches of the pine trees shown in Figure 3a.

In a previous study of the model on (001) substrates, enlarged images of the dendrites showed that their surfaces are mostly formed by (111) facets.^40^ The similar morphologies obtained here indicate that the (111) plane is also dominant. The brightest regions of the top views in Figure 4b,c, which are the top surfaces of the dendrite tips, have the same shape and approximately the same size as the patterns of the corresponding horizontal cross sections. Since these cross sections are parallel to the (111) plane, this is further evidence for the dominant (111) orientation of the dendrite surface.

When *P* increases to 0.4 (Figure 4c), the dendrites have much thicker branches, and they are predominantly oriented in the direction perpendicular to the (111) substrate. The top view suggests a flower-like organization where the petals have several orientations and rounded borders. This rounded morphology is reasonable for large *P* values because the differences in the hop probabilities are small for atoms with different numbers *n* of NNs (eq 1). However, Figures S1–S7 of the Supporting Information show that it is characteristic only of a narrow range of parameters with *P* ≈ 0.4 and *G* ≥ 2 × 10^3^. For *P* = 0.5 and *G* ≥ 2 × 10^4^, the growth of rounded columns with no branching is observed [Figures S6 and S7 of the Supporting Information]; similar features were formerly observed in SC lattices in these conditions.^55^

### Dendrite Morphology with FCC(110) Substrates

**Figure 5:**
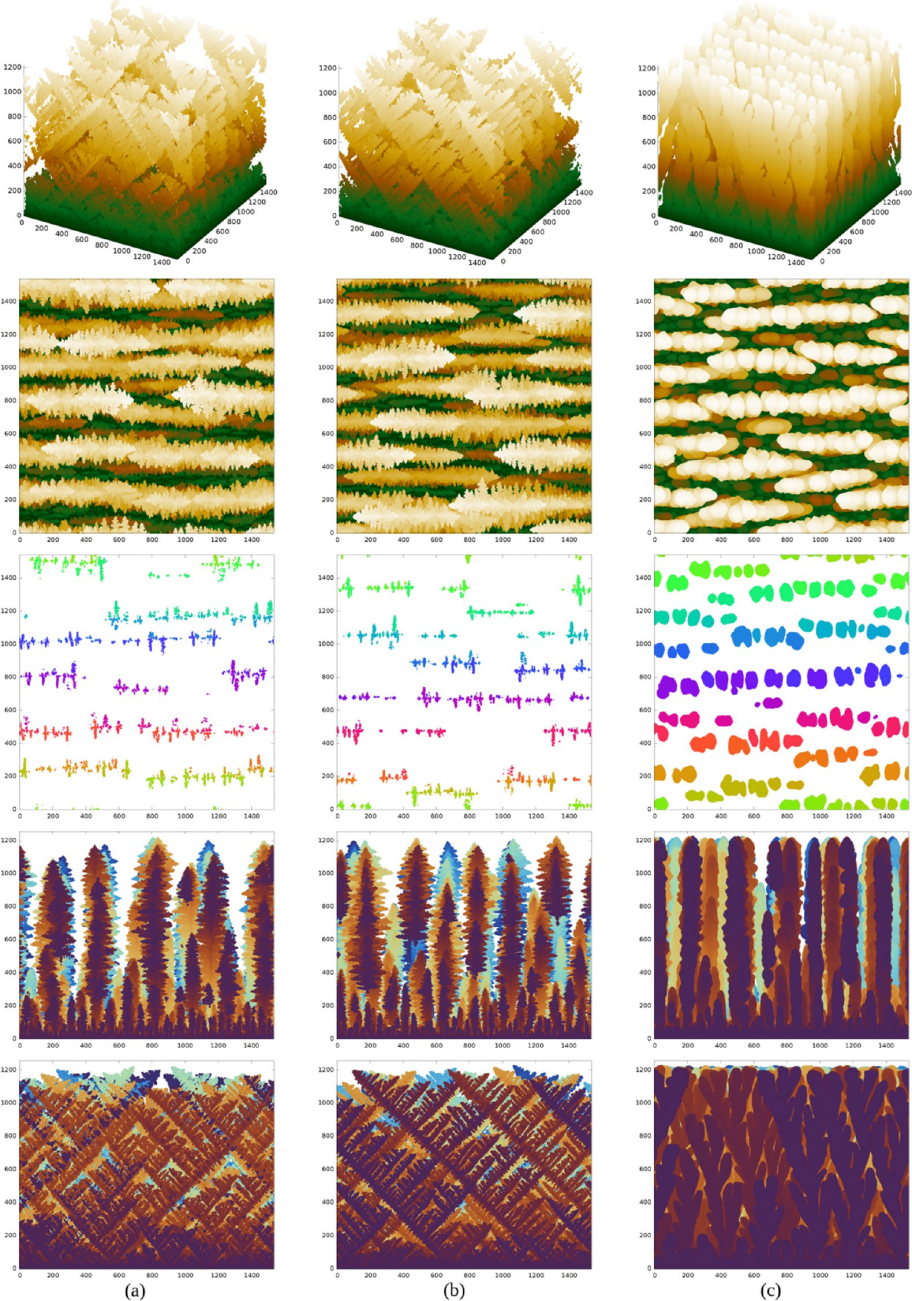
From top to bottom, perspective views, top views, and cross sections at ≈2/3 of the maximal height, and two lateral views of deposits grown on FCC(110) substrates with (a) *G* = 5 × 10^3^, *P* = 0.1; (b) *G* = 10^4^, *P* = 0.1; and (c) *G* = 10^4^, *P* = 0.4. All lengths are in units of the lattice constant.

Figure 5a–c shows views and cross sections of deposits grown with (*G* = 5 × 10^3^, *P* = 0.1), (*G* = 10^4^, *P* = 0.1), and (*G* = 10^4^, *P* = 0.4), respectively. Figures S8–S14 of the Supporting Information show images of deposits grown with 500 ≤ *G* ≤ 2 × 10^4^ and 0.025 ≤ *P* ≤ 0.5.

For *P* = 0.025 or *G* = 500, we also observe disordered branches with a DLA-like shape because the mobility of the adsorbed atoms is very low (see Figures S8–S14).

For *P* = 0.1, the top and lateral views in Figure 5a,b show dendrites with the same pine tree shape observed with other substrate orientations. The trunks grow in the directions [100] and [010]. The secondary and tertiary branches are mostly aligned with these directions, although small secondary branches also appear in the [−1,0,0] and [0,–1,0] directions, where the incidence of cations is shadowed by the branches of higher orders. Figures S8–S14 show that these organized patterns are observed up to a maximal value *P* = 0.3 and for a minimal value of *P* ranging from 0.2 for *G* = 10^3^ to 0.05 for *G* = 2 × 10^4^. This also means that these dendrites are formed if the mobility of the adsorbed atoms is high at configurations with a small coordination *n* (*GP*^*n*–1^ ≳ 1) and is low when the coordination is large (*GP*^*n*–1^ ≪ 1).

For *P* = 0.4 (Figure 5c), the branches are thicker, and their orientations change to a dominant propagation perpendicular to the substrate. The same is observed for other values of *G* ≥ 10^3^ and *P* ≥ 0.4 (Figures S13 and S14). With this substrate orientation, we do not observe the flower-like shape shown for a narrow parameter range when the substrate was (111).

### Dendrite Morphology with the HCP(0001) Substrate

**Figure 6:**
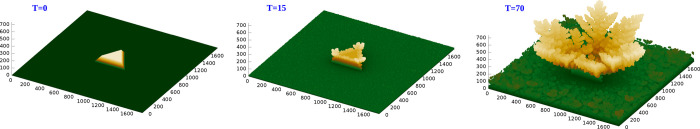
Images of a deposit grown in an HCP lattice with a truncated pyramid on the (0001) substrate and with *G* = 10^3^, *P* = 0.2. In all panels, the sequence of colors with increasing heights is roughly dark green, light green, brown, tan, cream, and white. All lengths are in units of the lattice constant and *T* is the nominal thickness.

In order to understand the details of the growth in this geometry, Figure 6 shows a typical evolution of the deposition with the (0001) plane patterned with a truncated pyramid (*T* = 0). The deposition preferentially occurs near the 3 vertices of the pyramid due to a tip effect, and the deposit in each of the vertices has a leaf-like shape (*T* = 15). However, at a longer deposition time (*T* = 70), six long trunks with secondary and tertiary branches are formed. They follow the hexagonal symmetry of the HCP lattice.

**Figure 7:**
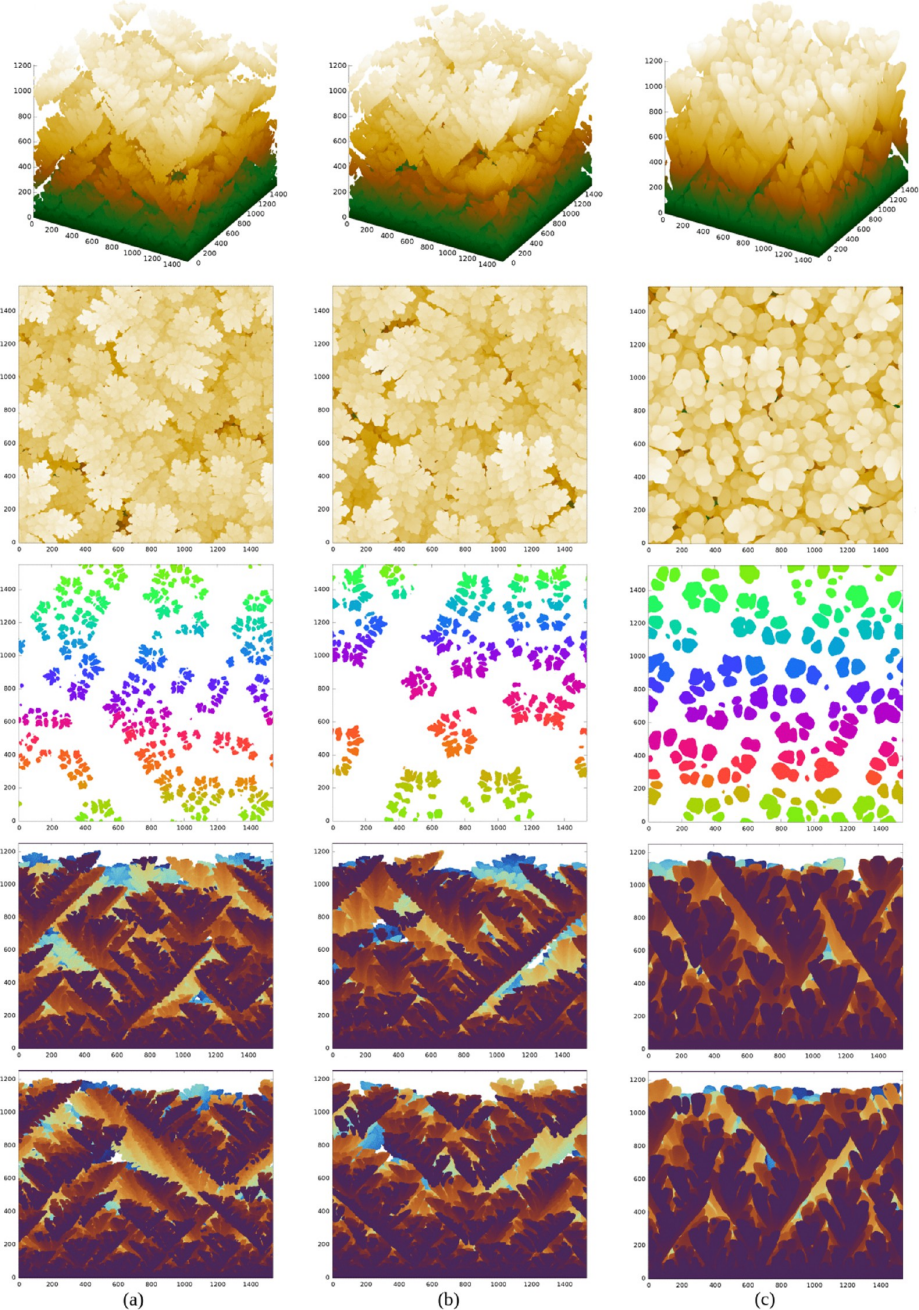
From top to bottom, perspective views, top views, and cross sections at ≈2/3 of the maximal height, and two lateral views of deposits grown on HCP(0001) substrates with (a) *G* = 10^4^, *P* = 0.2; (b) *G* = 2 × 10^4^, *P* = 0.2; (c) *G* = 10^4^, *P* = 0.4. All lengths are in units of the lattice constant.

Figure 7a–c shows views and cross sections of large deposits grown without patterning of the (0001) electrode and with parameters (*G* = 10^4^, *P* = 0.2), (*G* = 2 × 10^4^, *P* = 0.2), and (*G* = 10^4^, *P* = 0.4), respectively. Figures S15–S21 of the Supporting Information show similar images of deposits grown with 500 ≤ *G* ≤ 5 × 10^4^ and 0.025 ≤ *P* ≤ 0.5.

The smallest values of *G* and *P* lead to the formation of DLA-like patterns, similar to what is observed in the other lattices (see Figures S15–S21). However, Figure 7a,b show the growth of dendrites with a leaf-like shape following the hexagonal symmetry of the substrate. The similarity between the horizontal cross sections and the brightest parts of the top views indicates that the top parts of the dendrites are predominantly in the lowest-energy configuration (0001). The trunks propagate in directions that form an angle with the (0001) substrate and have projections on that substrate in the direction [101̅0] and in its five equivalent directions. Since the growth benefits the formation of low-energy surfaces, the dendrite surfaces are expected to be predominantly (101̅0) planes and equivalent ones, whose adatoms have *n* = 8 NNs (5 on the surface and 3 in the inside part of the branch). The parameter range in which these features are observed also obeys the conditions of *GP*^*n*–1^ ≳ 1 for the low coordinations (typically *n* ≤ 4) and *GP*^*n*–1^ ≪ 1 for the highest coordinations (typically *n* ≥ 7).

Figure 7c shows that, for large *P*, the vertical growth (perpendicular to the basal plane) is favored, and the dendrites have a flower-like shape. This is similar to the growth in the FCC(111) substrates but now a sixfold symmetry if fully developed.

### Dendrite Width

**Figure 8:**
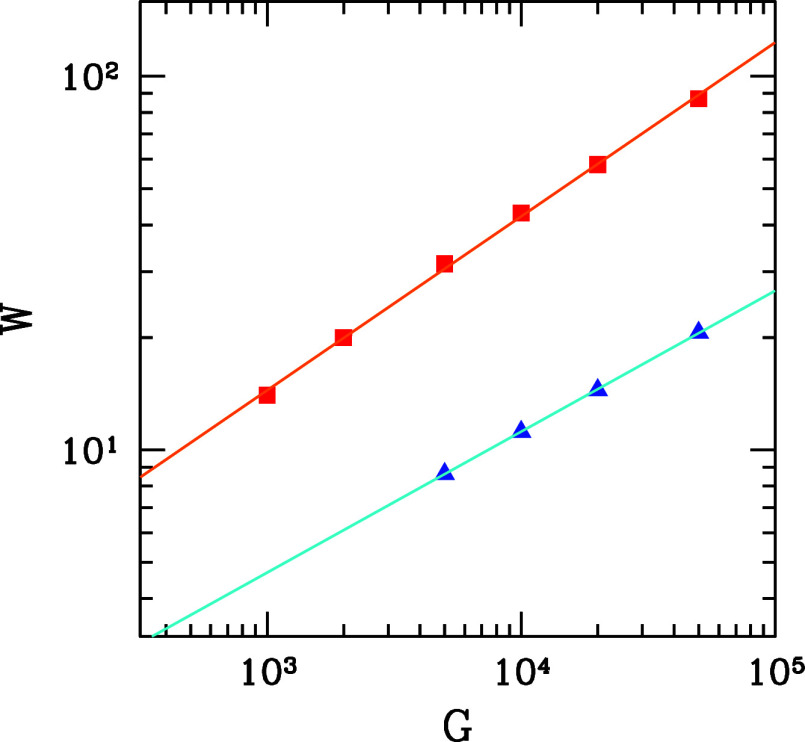
Average dendrite width in growth on FCC(111) substrates as a function of the parameter *G* for *P* = 0.1 (blue triangles) and *P* = 0.3 (red squares), with the linear fits of each data set. The width is given in units of the lattice constant.

Figure 8 shows the average dendrite width *W* as a function of *G* obtained in the growth on the FCC(111) substrate. The data for *P* = 0.1 and *P* = 0.3 are separately shown and are consistent with scalings with exponents of 0.38 and 0.47, respectively. The latter is close to the exponent 0.5 of the relation between the root mean square displacement of a random walker and the number of hops. This is consistent with the diffusive relaxation being responsible for shaping the dendrites. However, the results for *P* = 0.1 show deviations from this trend for much smaller values of *W*.

**Figure 9:**
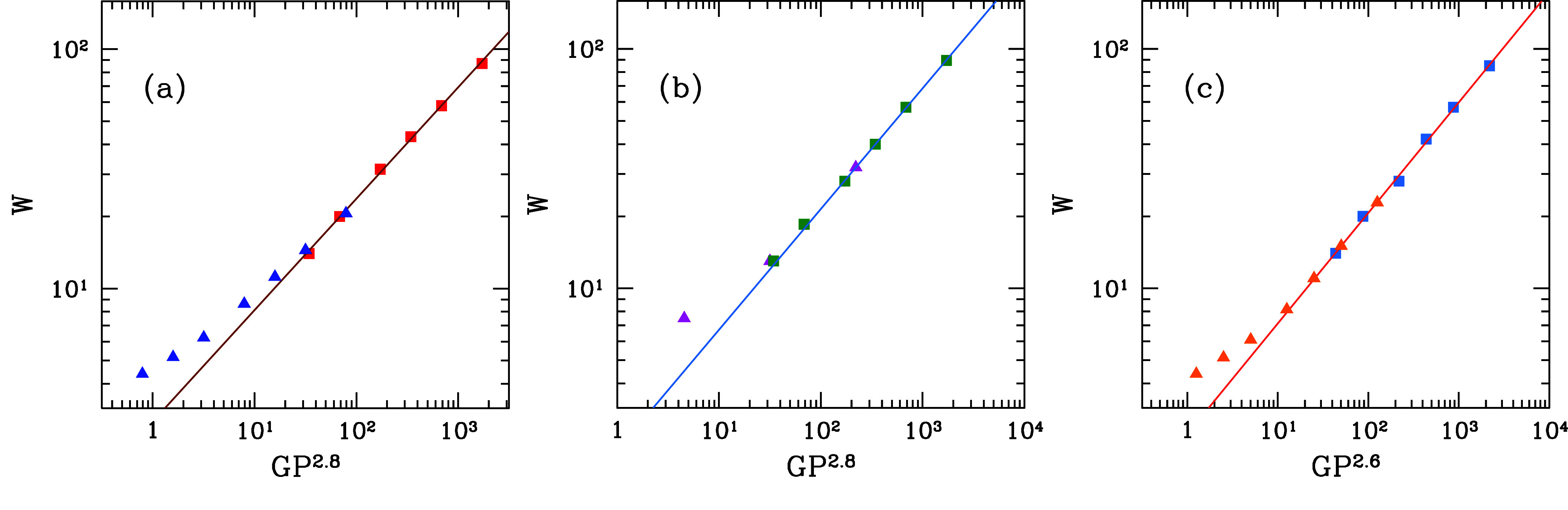
Average dendrite width as a function of scaled variables of *G* and *P*: (a) the same data as in Figure 8 for growth on FCC(111) substrates; (b) growth on FCC(110) substrates with *P* ≤ 0.2 (magenta triangles) and *P* = 0.3 (green squares); (c) growth on HCP(0001) substrates with *P* = 0.1 (orange triangles) and *P* = 0.3 (blue squares). The straight lines are linear fits of the data with scaling variables (abscissas) ≳10^2^. The widths are in units of the lattice constant.

The large difference in the values of *W* obtained for values of *P* that differ by a relatively small factor (eq 3), shown in Figure 8, indicates the need to search for a dependence on the model parameters that privileges the role of *P*. Figure 9a shows *W* as a function of scaling variable *GP*^2.8^, which leads to a good data collapse, with the largest widths for *P* = 0.1 and the smallest widths for *P* = 0.3. The linear fit for *GP*^2.8^ ≳ 10^2^ has a slope close to 0.5.

For growth on FCC(110) substrates, we also obtain a good data collapse of the average width for several *G* and *P* values with scaling variable *GP*^2.8^, as shown in Figure 9b. Now the fit for *GP*^2.8^ ≳ 10^2^ considers only data with *P* = 0.2 and 0.3 and has a slope of 0.50. For growth on the HCP lattice with (0001) substrates, Figure 9c shows that *W* as a function of *GP*^2.6^ provides the best collapse of data with *P* = 0.1 and *P* = 0.3. A linear fit with a slope of 0.46 is obtained for *GP*^2.6^ > 10^2^.

A similar analysis was formerly performed with the data from growth on FCC(001) substrates and provided a slope of 0.4 for the logarithmic plot of *W* versus *GP*^3^.^40^ In SC lattices, the appropriate scaling variable was shown to be *GP*,^62^ and the corresponding logarithmic plots of the dendrite widths had slopes between 0.38 and 0.43.^55^

### Controls of Dendrite Shape and Size

**Figure 10:**
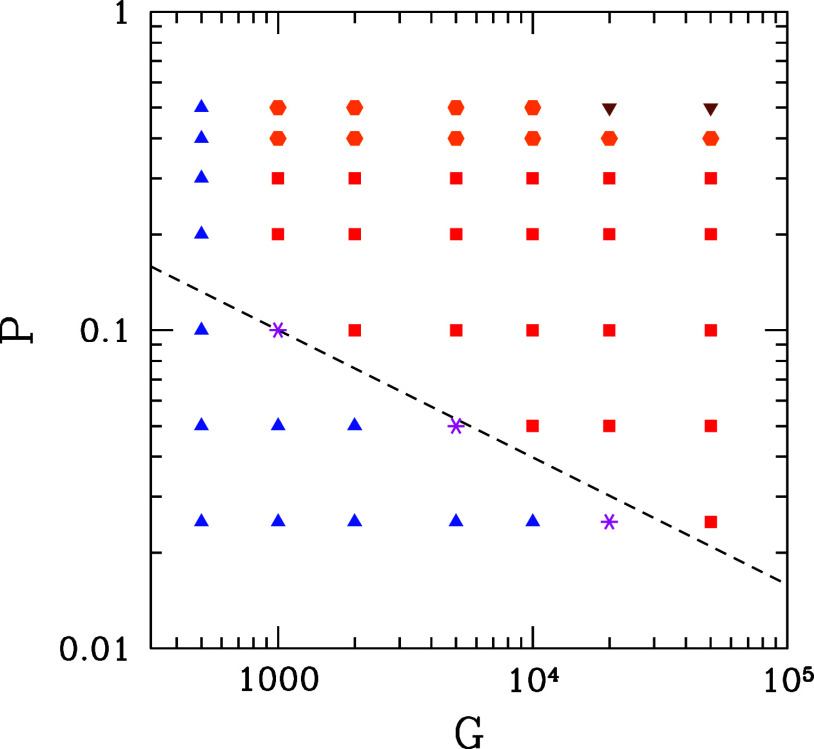
Morphology diagram for FCC(111) substrates: DLA-like deposits (blue triangles), transition patterns (magenta asterisks), dendritic deposits (red squares), flower-like patterns (orange hexagons), or columnar films (brown inverted triangles). The dashed line has *GP*^2.5^ ≈ 3.

The diagram in Figure 10 shows the morphologies of the deposits obtained in simulations with FCC(111) substrates (electrodes) for each parameter set (*G*,*P*): DLA-like, transition, dendritic, flower-like, or columnar. Similar diagrams are obtained in other lattices and other substrate orientations, as formerly shown in the SC lattice.^39^ The flower-like shapes may also be interpreted as transition patterns, i.e., intermediate between dendrites (hierarchically organized) and the columnar structures (vertically aligned). The dendritic patterns, which are the focus of the present study, generally appear above the dashed line of Figure 10, in which *GP*^2.5^ ≳ 3.

The crystallography of the dendrites in the FCC lattice depends on the substrate orientation relative to the direction of the cation flux: the (111) surfaces are always dominant and the propagation of the branches always occurs in the directions with the smallest Miller indices ([001] and equivalent ones), so the angles between branches of different orders are always of 90°. The dendrites may develop apparently different shapes at large scales depending on the substrate and the angle of visualization. This occurs when their trunks grow in a direction different from that of the gradient of cation concentration in the electrolyte (which is perpendicular to the substrate in all simulations). In this case, the growth of some secondary or tertiary branches is shadowed, so that the pine tree shape may change into, for example, a leaf-like shape.

At long times and large length scales, the diffusive cation flux and shadowing effects lead to unstable growth and may produce the different patterns summarized in Figure 10. However, surface diffusion of the mobile atoms organizes the deposit at short times and short length scales. The pine trees (dendrites) appear if the model parameters obey the relations *GP*^*n*–1^ ≳ 1 for mobile atoms with low coordinations (typically *n* ≤ 4) and *GP*^*n*–1^ ≪ 1 for atoms with high coordinations (typically *n* ≥ 7). The first relation allows the mobile atoms to spread to distant points (at the nanoscale) when they are on low-energy planes [e.g., (111) or (001)] and the second relation ensures that low-energy patterns are stable.

An important assumption of the model is the independence of the hopping probability of eq 1 from the spatial orientation of the NNs of the mobile atom. For instance, if the mobile atom has *n* = 3 NNs, these NNs may be in a (111) plane (forming a regular tetrahedron with the mobile atom) or these NNs may be in planes with other orientations, but *P*_hop_ = *P*^2^ in both cases. Considering that eq 2 is a physically plausible form for the probability *P*, it implies that the activation energy for hopping is additive over the NNs of the mobile atom. The pine tree morphology is related to this assumption of isotropic interactions with the NNs. If the mobility of the atoms was also dependent on the particular orientation of the NNs (a case not studied here), then the dendrite crystallography may change.

In the HCP lattice, the leaf-like dendrites follow the hexagonal symmetry and differ from those of the FCC lattice. Differences are also observed in dendrites grown in SC lattices.^39,62^

The analysis of the dendrite widths in these lattices provided relations with the diffusion length of the adsorbed atoms, which are represented by the mobile atoms in the model. In the scalings of Figure 9a–c for FCC and HCP lattices, exponents 2.8 and 2.6 were obtained for the dendrite widths, respectively. The morphological diagram in Figure 10 shows the approximate relation *GP*^2.5^ ≳ 3 for observing the dendrites, which is consistent with widths *W* ≫ 1. However, eq 1 for the hop probability involves only integer exponents of *P*. The closest integer value in these scaling relations is 3, so an approximate scaling for the dendrite width is proposed as follows
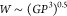
3In the SC lattice, a previous study^62^ showed a scaling of *W* with (*GP*)^0.5^.

Mobile atoms on the surfaces of FCC and HCP dendrites have *n* = 3 NNs, so *P*_hop_ = *P*^2^, whereas mobile atoms on the lowest-energy surfaces of SC lattices [(001)] have *P*_hop_ = 1. Thus, *GP*^3^ ≫ 1 in the FCC lattice means that a mobile atom on a (111) plane can execute a large number of hops even if it has an NN on the same plane [besides the 3 NNs of the (111) plane]. The same is applicable to mobile atoms on (001) planes of an SC lattice when *GP* ≫ 1. These atoms can easily reach the borders of the low-energy planes, find positions for aggregation there, and enlarge their areas until the widths reach the values in eq 3. The same applies to surfaces of dendrites in the HCP lattice.

Thus, two general conclusions can be drawn from these scaling relations. First, the dendrite width is on the order of the diffusion length of an atom from its production (by cation reduction) to its permanent crystallization. This may be used to understand microscopic features of electrodeposited patterns even without specific modeling. Second, the high mobility of the atoms on low-energy planes permits these atoms to easily reach the borders of these planes and explains the dominant low-energy orientations of the dendrite surfaces.

### Dendrite Orientation and Physicochemical Properties

In FCC materials, the first important effect of the electrode orientation is the suppression of the growth of secondary branches when the orientation is (111) or (110). This implies a reduction in the surface area of the dendrites, which may reduce the rate of catalytic reactions on their surfaces. For these reactions and for other applications in which the exposed area at the molecular scale is important, the best growth conditions are those that lead to the full development of secondary and higher-order branches.

The dendrite orientation may also have effects on the physicochemical properties of thin films if they are mechanically stable in the configuration in which they grow. For instance, the orientation changes the porous media formed between the dendrites. With an FCC(001) electrode, wide and vertically oriented pores are formed between the aligned nanotrees (Figures 2 and 3a), which is a configuration with visibly smaller pore tortuosity than the media formed between the nanotrees grown on (110) or (111) electrodes (Figures 4 and 5). Physical processes that depend on the diffusion of chemical species in these pores are then affected by these changes; the ionic conductivity is expected to be higher in the less tortuous porous media.

Importantly, these conclusions depend on the spatial organization of different dendrites but do not depend on their individual shapes. As explained above, the dendrite shape may change only if the energetics of the atomic interactions change.

### Comparison with Other Models

To our knowledge, other models of electrodeposition assumed that all adsorbed atoms of the crystal surfaces could simultaneously move, in a process usually termed collective adatom diffusion.^45−51^ If the adatom diffusion length is very small during the deposition time, randomly branched aggregates with a DLA shape are obtained;^23^ in some studies, they are termed fractal aggregates.^47^ If the adatom diffusion becomes important, compact films or columnar deposits may be obtained.^45−51^

The collective diffusion allows the surfaces to converge to their lowest-energy configuration, but the randomness of the deposition flux continuously works against that convergence. If the cation flux is diffusive, these cations are preferentially reduced in the upper parts of the deposits, while the bottom parts are not accessible and evolve only through adatom diffusion. Thus, any pattern formed at these bottom parts will be destroyed by this relaxation process, which brings the surfaces to low-energy configurations (at least in short length scales). This would be the case, for instance, of the nanosized secondary and tertiary branches of the dendrites formed in our simulations.

Instead, the electrodeposition model considered here assumes that each adsorbed atom moves only for a limited time interval before crystallization. For this reason, the dendritic patterns formed in all parts of the deposits are stable. This model accounts for the TEM observations that the tips of silver dendrites have crystalline cores surrounded by amorphous layers with thicknesses of a few nanometers and that the cores grow by the crystallization of these layers.^25^ There is deposition of new material at the interface between the amorphous layer and the electrolyte, but this process does not affect the stable crystalline core.

Other important assumptions of our model are the purely diffusive cation flux, which results from a concentration gradient near the film surface and the rapid electrochemical reaction. The first assumption is reasonable for a supported electrolyte, in which inert ions reduce the electric field and suppress ion migration,^64^ and is consistent with former applications of the model.^40,41^ The introduction of an electric field bias in the model may have two consequences: (i) allow the cations to reach deeper parts of the deposits, which reduces the shadowing effect; (ii) increase the tip effect due to higher electric fields at protuberant points of the surface, which contribute to shadowing. Thus, the overall effect on the sizes of the dendrite branches is not trivial. Regarding the second assumption, a finite rate of the electrochemical reaction would allow the cations to reduce at the deepest points of the deposit (instead of the first contact) and would reduce the shadowing effect. However, this change will be restricted to a finite depth below the film surface, where the cations can penetrate.

### Comparison with Experiments

A transition from the growth of compact particles to dendrites with a pine tree shape was shown in KMC simulations of the model in FCC lattices and represented the transition of silver electrodeposits on gold nanoparticle-patterned HOPG substrates.^40^ However, this pine tree shape is also observed in gold electrodeposition. For instance, it is shown in high-resolution scanning electron microscopy (SEM) images of dendrites with nanosized secondary branches growing from microsized trunks^52^ and in a recent field-emission SEM image of dendrites grown on a carbon surface.^53^

In a narrow range of parameters, the simulations in FCC and HCP lattices also show flower-like dendrites (at least for some angles of visualization) [see Figures 4c and 7c]. This is a feature of a large *P*, in which the diffusion of mobile atoms is weakly dependent on the local surface morphology of the crystal. The shape resembles that of gold eletrodeposits on modified indium–tin oxide (ITO) substrates (where the use of ITO without modification leads to DLA-like shapes)^65^ and of Ag and Ag–Cu alloys on Cu foil substrates.^1^ We are not aware of electrodeposition studies that show the nearly hexagonal flower-like patterns obtained here for the HCP geometry, but our results may be useful to anticipate the features of these deposits.

Instead, several studies show electrodeposited dendrites with shapes different from those obtained here, such as the feather-like shapes of silver dendrites that spread in the [112] direction.^66^ A possible reason for this difference is a deviation from the isotropic interaction rule of the present model, which is implicit in eq 1 and naturally favors surface orientations with the largest numbers of NNs at short scales, independent of the spatial orientation of these NNs. These deviations are important, for instance, in modeling homoepitaxial growth from vapor sources because activation energies for diffusion on low-energy planes have different in-plane and out-of-plane contributions per NN (although in-plane additivity is frequently assumed).^44^

Interestingly, some morphological transitions observed here seem to be equivalent to those observed in the growth of two-dimensional materials. For instance, graphene flakes grown on Ag(111) may have a dendritic shape with a hexagonal symmetry^67^ similar to the electrodeposits on HCP(0001) substrates, as shown in Figure 7a–c. The transitions between that shape and compact flakes are shown in phase-field simulations,^68^ in parallel with the transitions to columnar structures observed here.

## Conclusions

We studied the morphology of electrodeposited dendrites in different lattices using a model that represents the interplay between the diffusive cation flux in the electrolyte and diffusion of the atoms after their adsorption. The diffusion of the adsorbed atoms is controlled by two parameters: *G*, which is the number of hop attempts, and *P*, which is the probability of accepting each attempt per occupied the NN at the current position. Thus, the adatom easily moves in regions where *GP*^*n*–1^ ≫ 1, moves only in a close neighborhood if *GP*^*n*–1^ ∼ 1, and is frozen at points where *GP*^*n*–1^ ≪ 1. KMC simulations were performed on FCC and HCP lattices, considering planar electrodes (substrates) and possible nucleation at all of their sites. In the FCC lattice, substrate orientations (111) and (110) were considered, and the results were compared with previous simulations with (001) substrates;^40^ in the HCP lattice, substrate orientation (0001) was considered; previous results in SC lattices with (001) substrates were also addressed.^39^

The electrodeposition with the FCC(001) substrate forms dendrites with a pine tree shape whose trunk is oriented perpendicular to the substrate and whose higher-order branches are perpendicular to those of the orders immediately below. With the FCC(110) substrate, the trunk orientations change to directions [100] and [010] and one of the secondary branches does not grow due to shadowing of the incoming cation flux by these trunks. With the FCC(111) substrate, two neighboring secondary branches are suppressed by shadowing, which leads to the growth of dendrites with a leaf-like shape and a triangular symmetry of the trunk propagation (in [100], [010], and [001] directions). Despite the different visual impressions of these dendrites, caused by shadowing and choices of image orientation, they always propagate in the directions with the smallest Miller indices, and their surfaces are predominantly of (111) orientation. The pine tree shape was formerly observed in silver electrodeposition on substrates patterned with gold nanoparticles^40^ and here we briefly noted that similar morphologies are shown in other studies with silver, gold, and Ag–Cu alloy electrodeposition. In the HCP lattice, the main difference is the hexagonal symmetry of the dendrites, which also have a leaf-like shape.

The hierarchically organized structures discussed above were obtained for broad ranges of parameters in which *GP*^*n*–1^ ≳ 1 for mobile atoms with coordinations *n* ≤ 4 and *GP*^*n*–1^ ≪ 1 for atoms with coordinations *n* ≥ 7. For a narrow range of large *P* (0.4–0.5), the local neighborhood of the adsorbed atom weakly affects its mobility. In these conditions, in both lattices, we observe the formation of rounded dendrites with a flower-like shape and trunks propagating mainly in the direction perpendicular to the electrode.

We also measured average dendrite widths from cross sections of the deposits parallel to the substrates. In the FCC and HCP lattices, these widths scale approximately as *GP*^3^, which is on the same order of magnitude as the diffusion length of the atoms with four NNs (*n* = 4) from adsorption to permanent incorporation to the crystal. These are typically the atoms on the lowest-energy surfaces of the dendrites with one NN on the same surface. Similar results are obtained in the SC lattice and confirm that dendrite widths may be taken as an estimate of the diffusion length of the adsorbed material.

## Data Availability

The data shown in this paper is available by request to the authors.

## Supporting Information Available

Images of deposits for several values of the model parameters (PDF)
