# Supplementary material for: Relaxation of the Adsorbed Material and Shadowing Effects on the Shape and Size of Electrodeposited Dendrites
Source: Langmuir. 2025 Apr 25;41(17):11068–80. doi: 10.1021/acs.langmuir.5c00740 (PMC12060280; doi:10.1021/acs.langmuir.5c00740)
Supplement: Supplementary file 1 — la5c00740_si_001.pdf [file la5c00740_si_001.pdf]

Supporting Information
Relaxation of the Adsorbed Material and Shadowing Effects on the Shape and Size of Electrodeposited Dendrites

Dung di Caprio[1], Abdelhafed Taleb[2], and Fábio D. A. Aarão Reis[3,*]

[1]PSL Research University, Chimie ParisTech - CNRS, Institut de Recherche de Chimie Paris, 75005, Paris, France, dung.di-caprio@chimieparistech.psl.fr

[2]PSL Research University, Chimie ParisTech - CNRS, Institut de Recherche de Chimie Paris, 75005, Paris, France, abdelhafed.taleb@upmc.fr

[3]Instituto de Física, Universidade Federal Fluminense, Avenida Litorânea s/n, 24210-340 Niterói, RJ, Brazil, fdaar@protonmail.com

[*]Corresponding author

List of Figures

S1 2
S2 3
S3 4
S4 5
S5 6
S6 7
S7 8
S8 9
S9 10
S10 11
S11 12
S12 13
S13 14
S14 15
S15 16
S16 17
S17 18
S18 19
S19 20
S20 21
S21 22

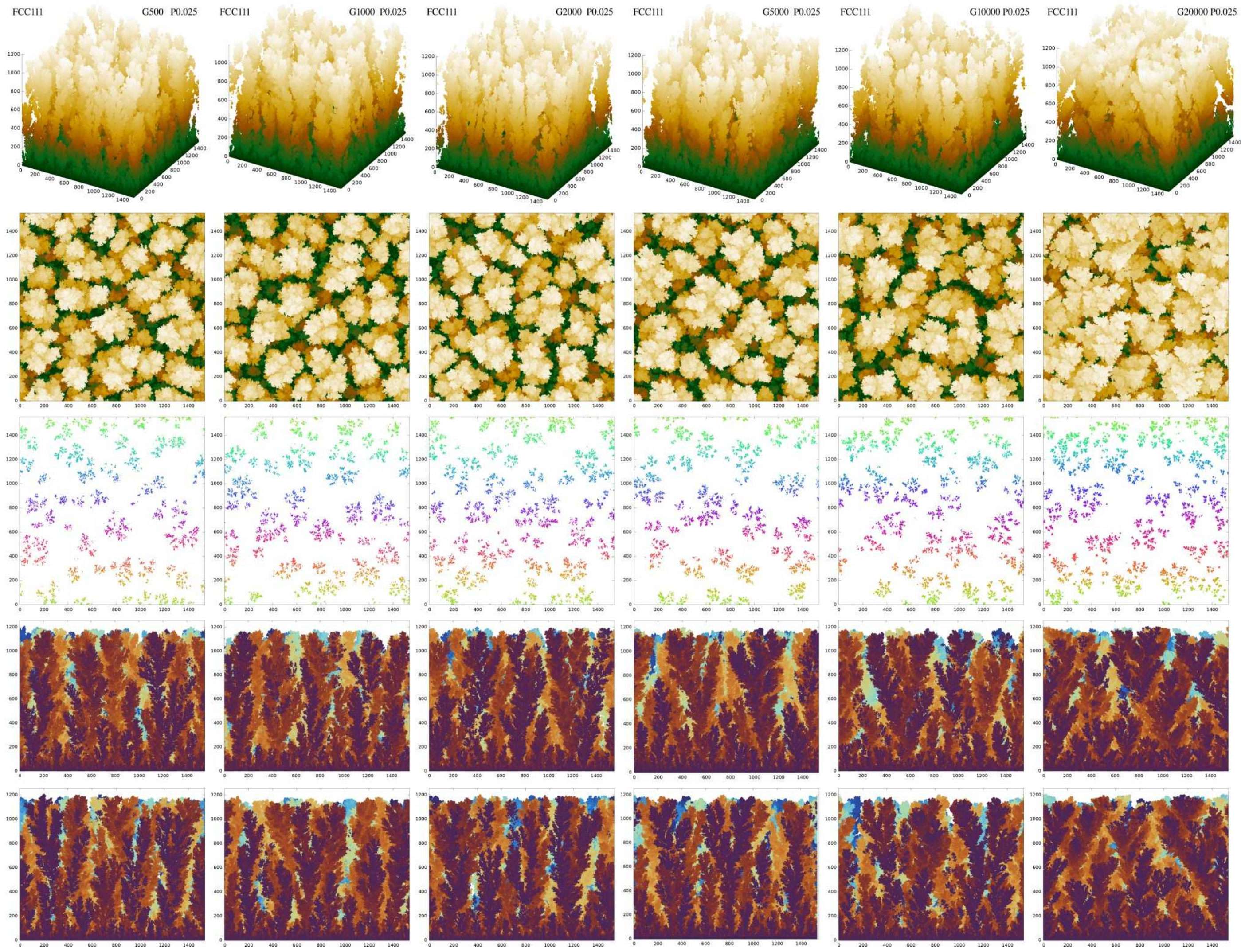

Figure S1: From top to bottom, perspective views, top views, cross sections at $\approx 2/3$ of the maximal height, and two lateral views of deposits grown on FCC(111) substrates with $P = 0.025$ and the indicated values of G.

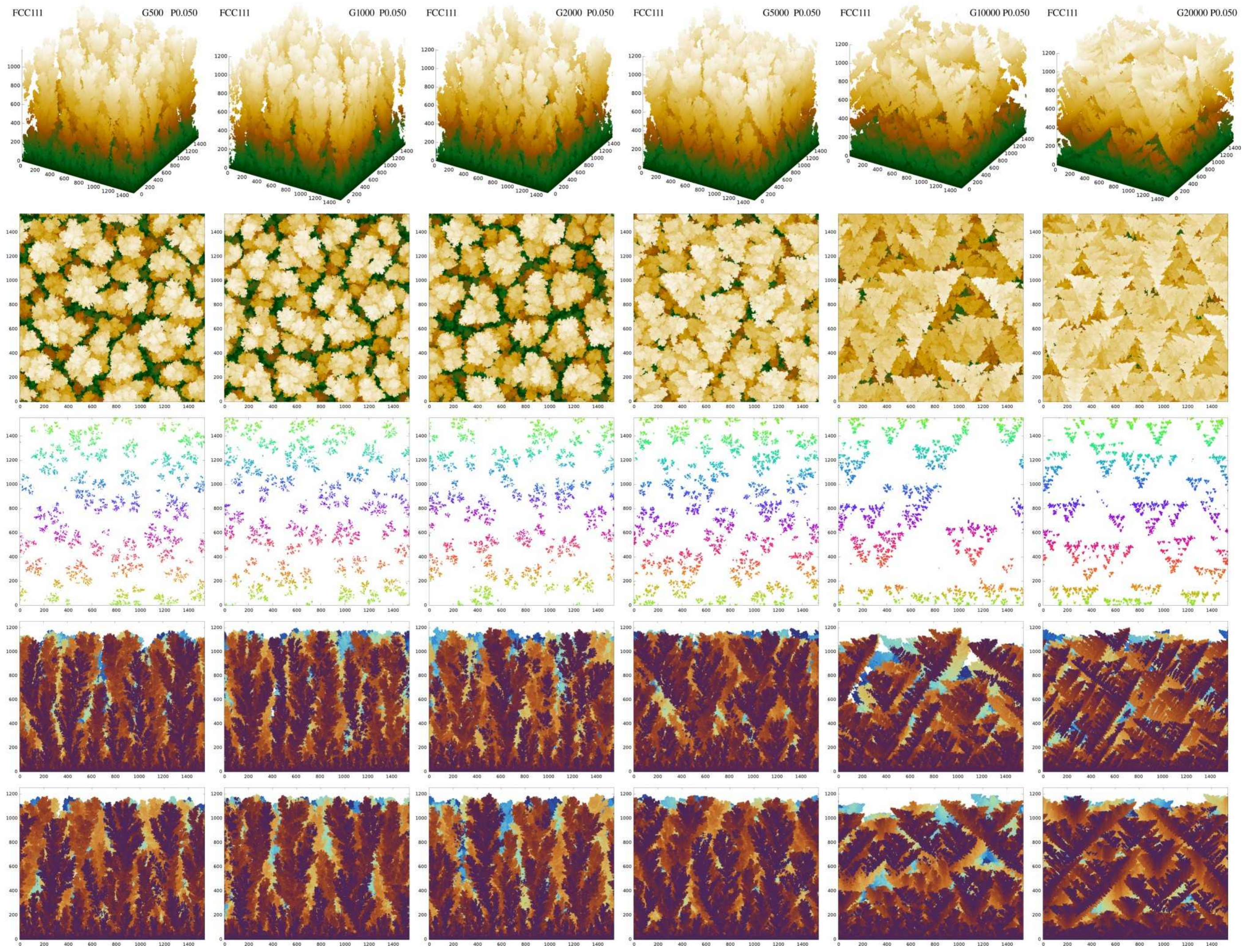

Figure S2: From top to bottom, perspective views, top views, cross sections at $\approx 2/3$ of the maximal height, and two lateral views of deposits grown on FCC(111) substrates with $P = 0.05$ and the indicated values of G.

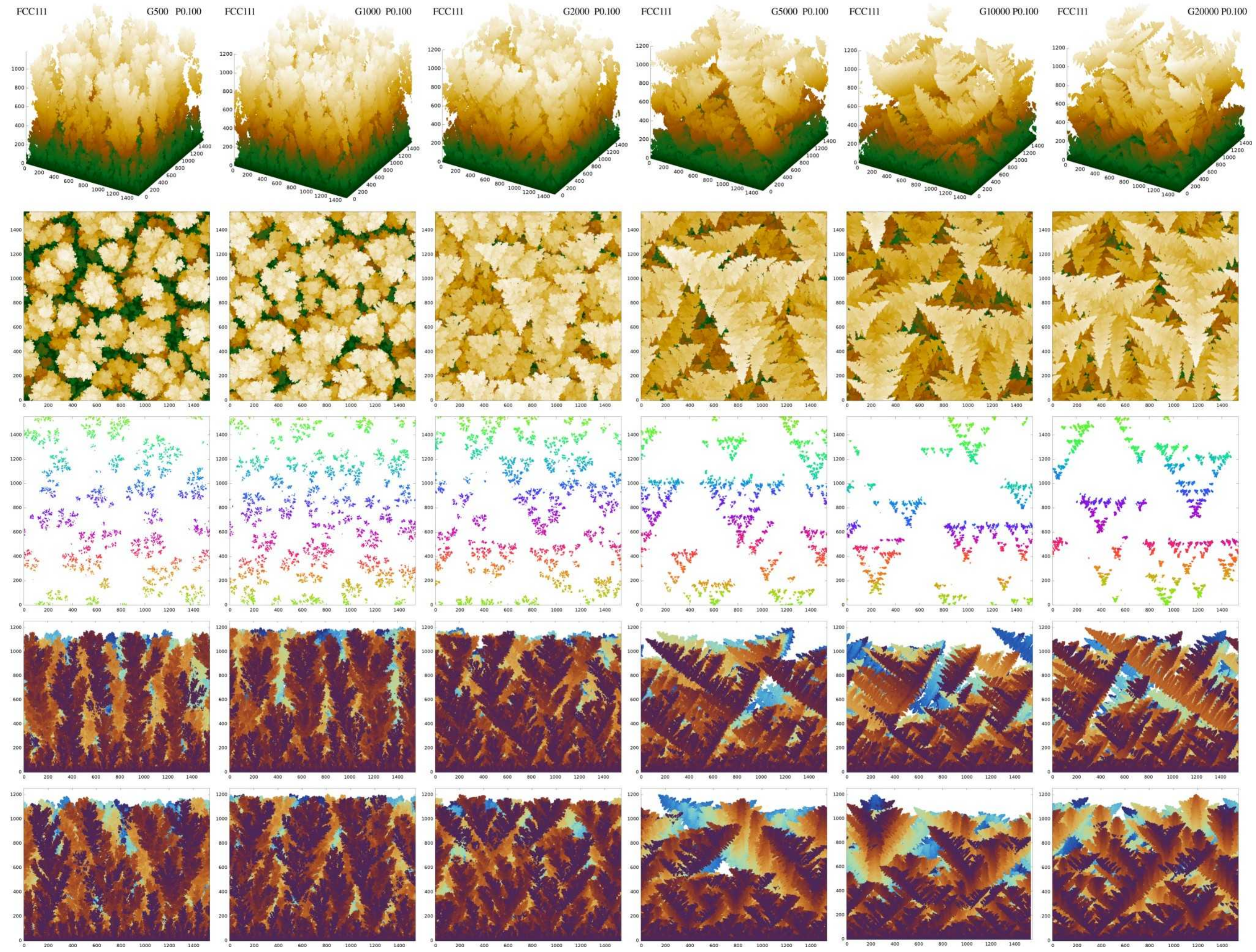

Figure S3: From top to bottom, perspective views, top views, cross sections at $\approx 2/3$ of the maximal height, and two lateral views of deposits grown on FCC(111) substrates with $P = 0.1$ and the indicated values of G.

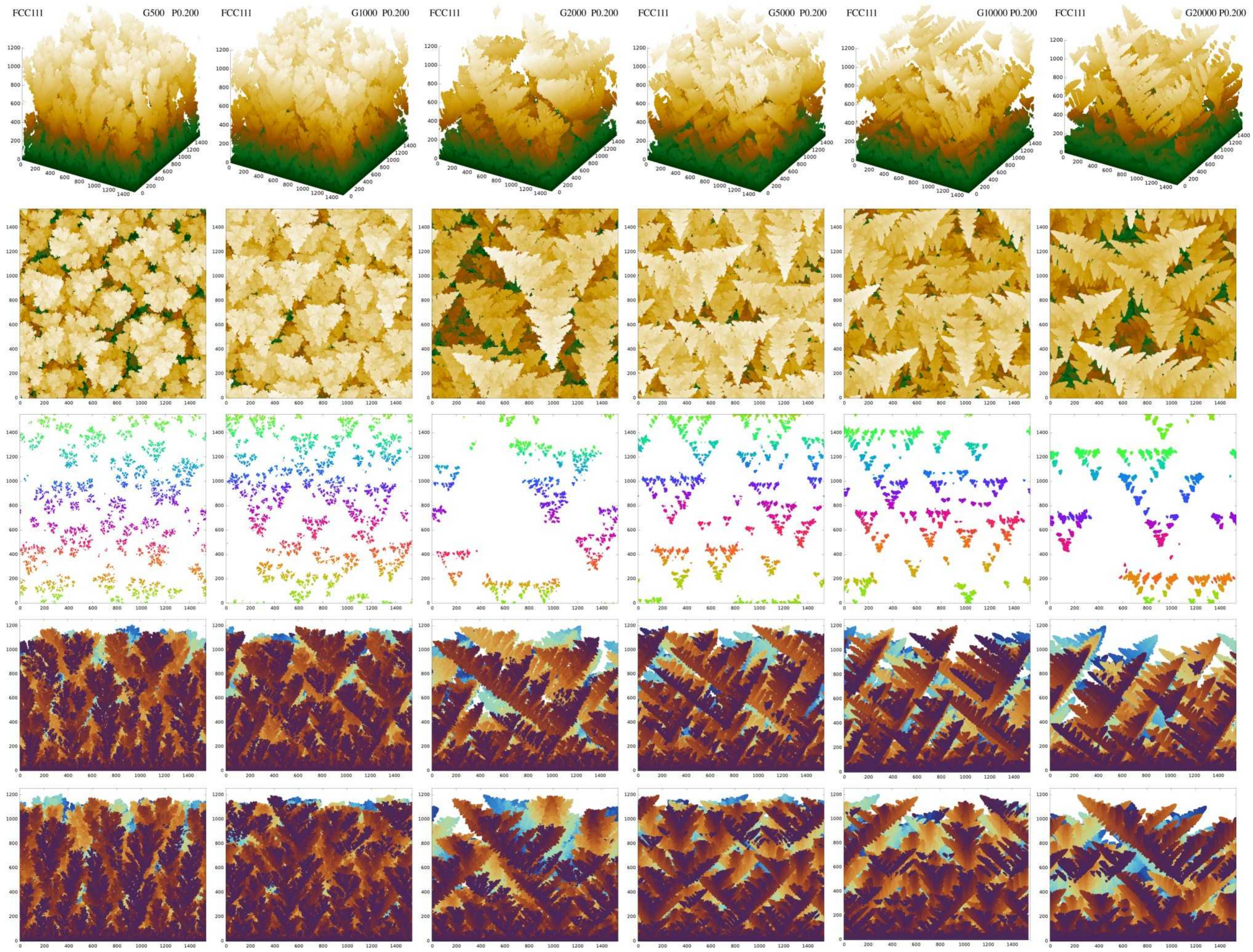

Figure S4: From top to bottom, perspective views, top views, cross sections at $\approx 2/3$ of the maximal height, and two lateral views of deposits grown on FCC(111) substrates with $P = 0.2$ and the indicated values of G.

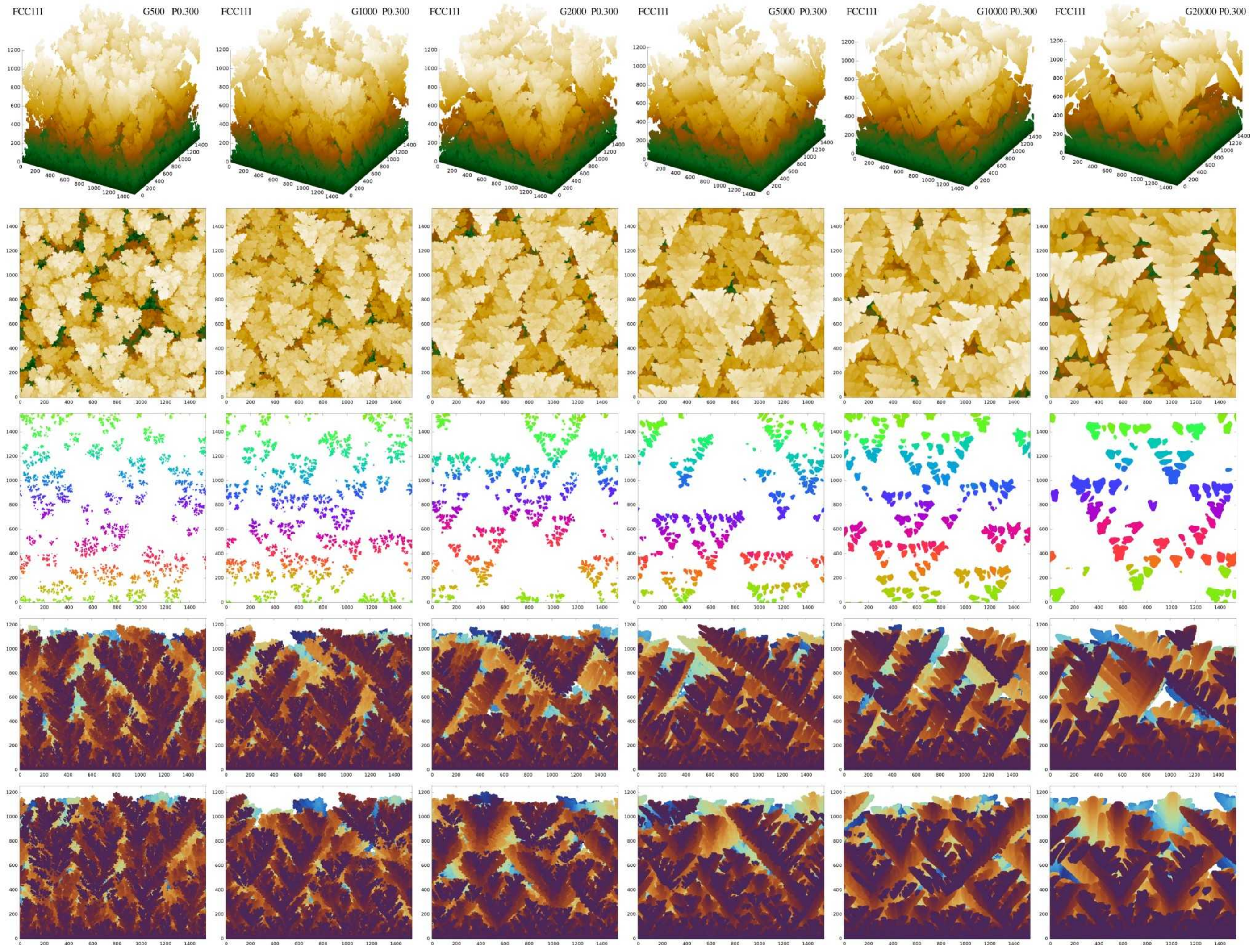

Figure S5: From top to bottom, perspective views, top views, cross sections at $\approx 2/3$ of the maximal height, and two lateral views of deposits grown on FCC(111) substrates with $P = 0.3$ and the indicated values of G.

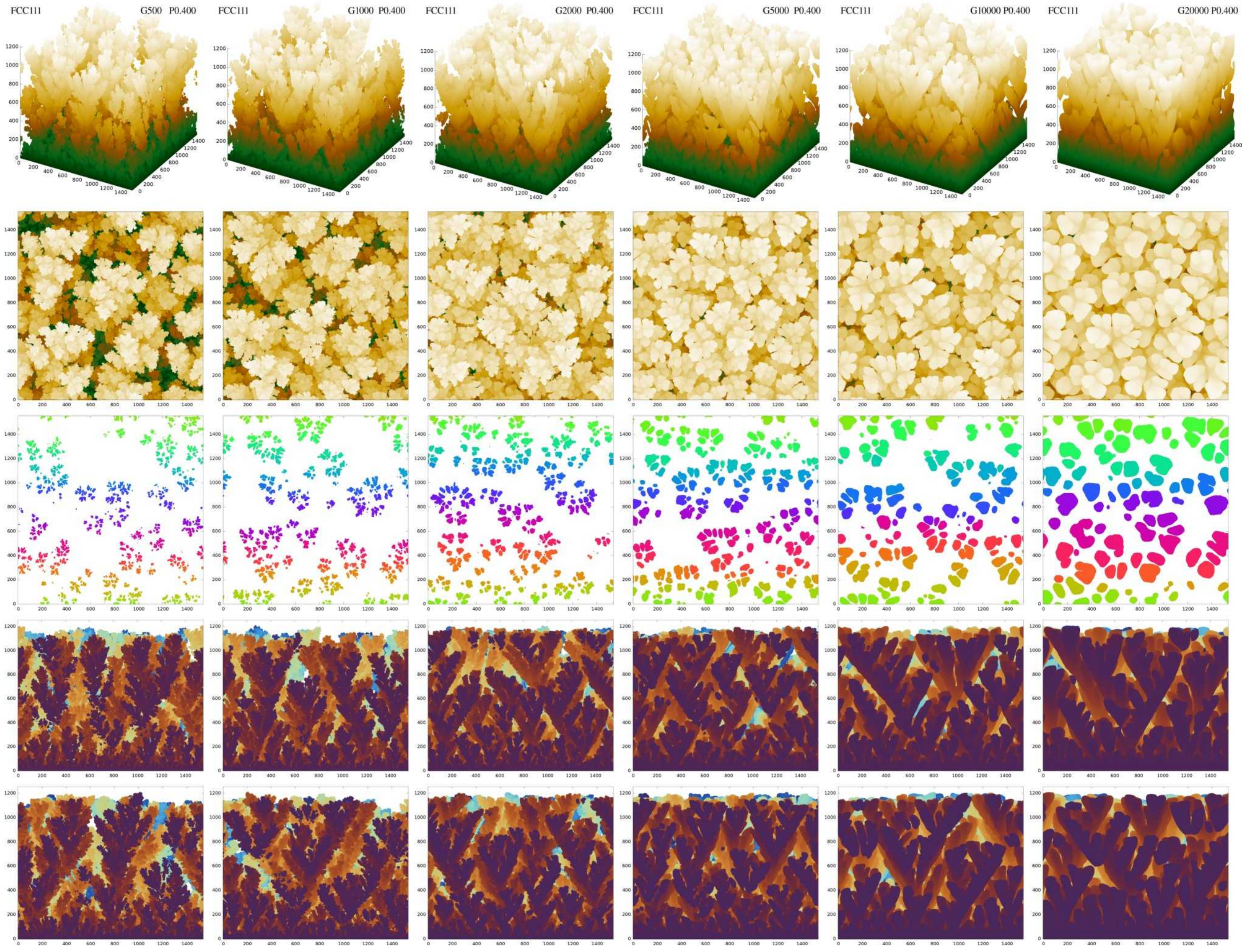

Figure S6: From top to bottom, perspective views, top views, cross sections at $\approx 2/3$ of the maximal height, and two lateral views of deposits grown on FCC(111) substrates with $P = 0.4$ and the indicated values of G.

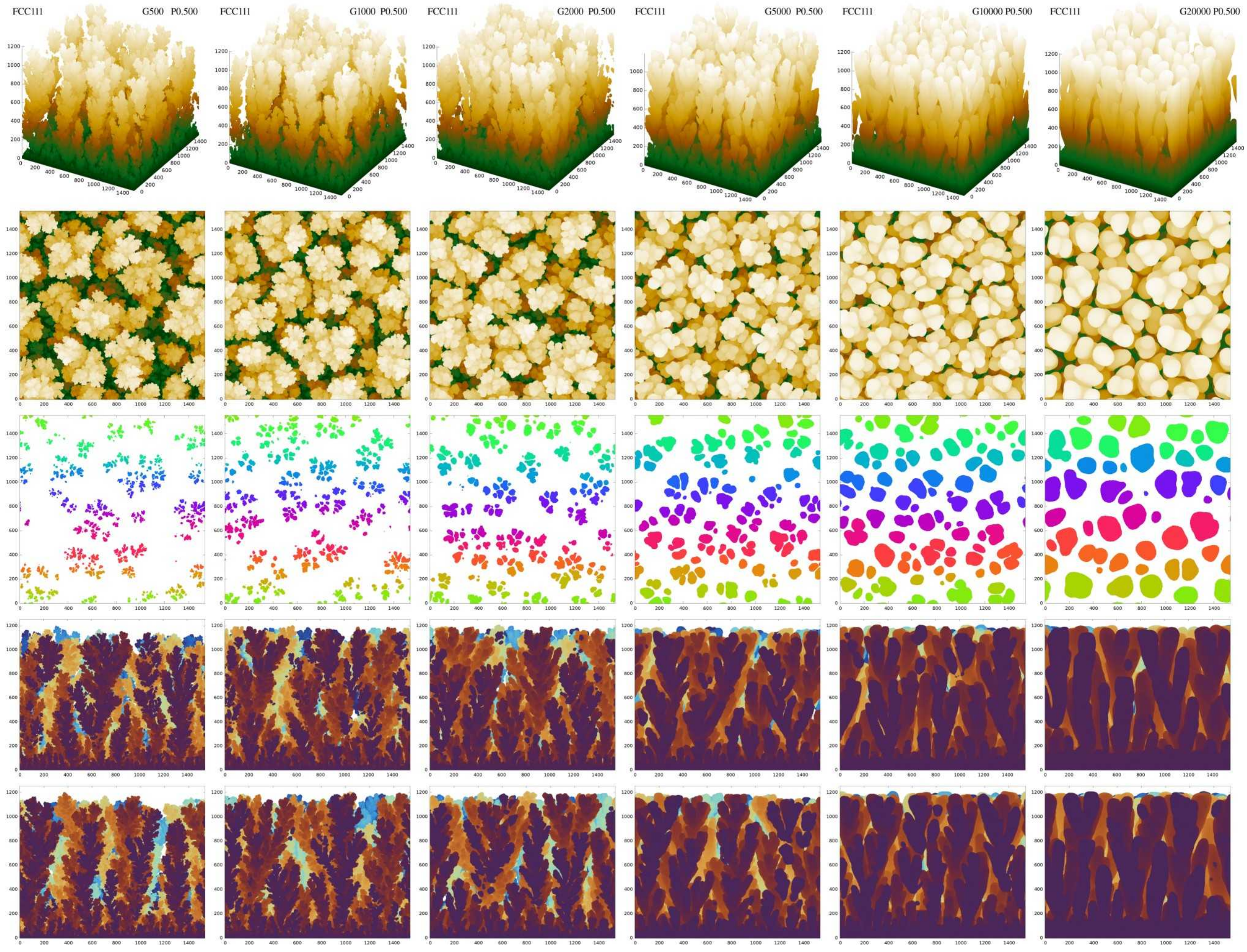

Figure S7: From top to bottom, perspective views, top views, cross sections at $\approx 2/3$ of the maximal height, and two lateral views of deposits grown on FCC(111) substrates with $P = 0.5$ and the indicated values of G.

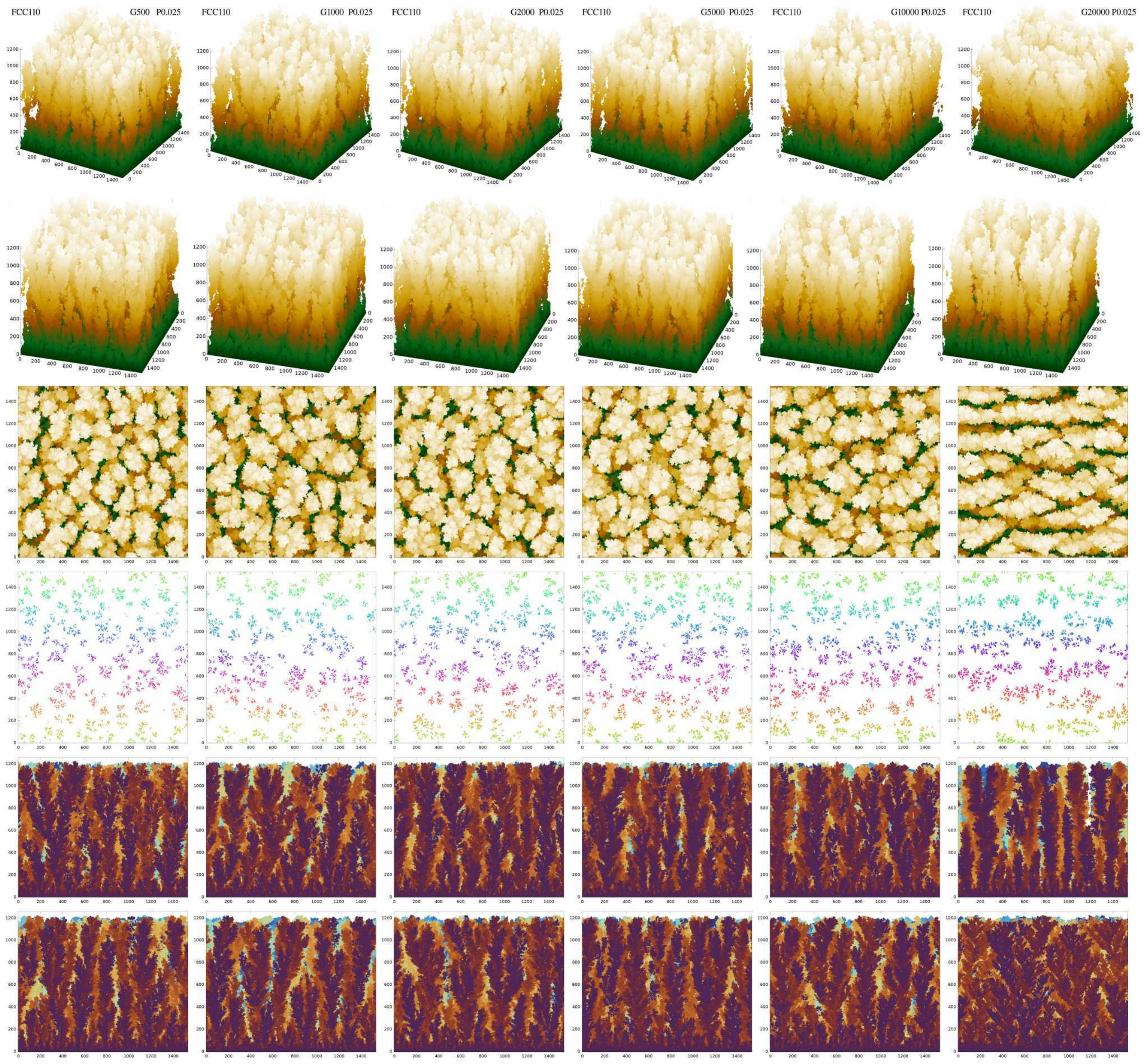

Figure S8: From top to bottom, perspective views, top views, cross sections at $\approx 2/3$ of the maximal height, and two lateral views of deposits grown on FCC(110) substrates with $P = 0.025$ and the indicated values of G.

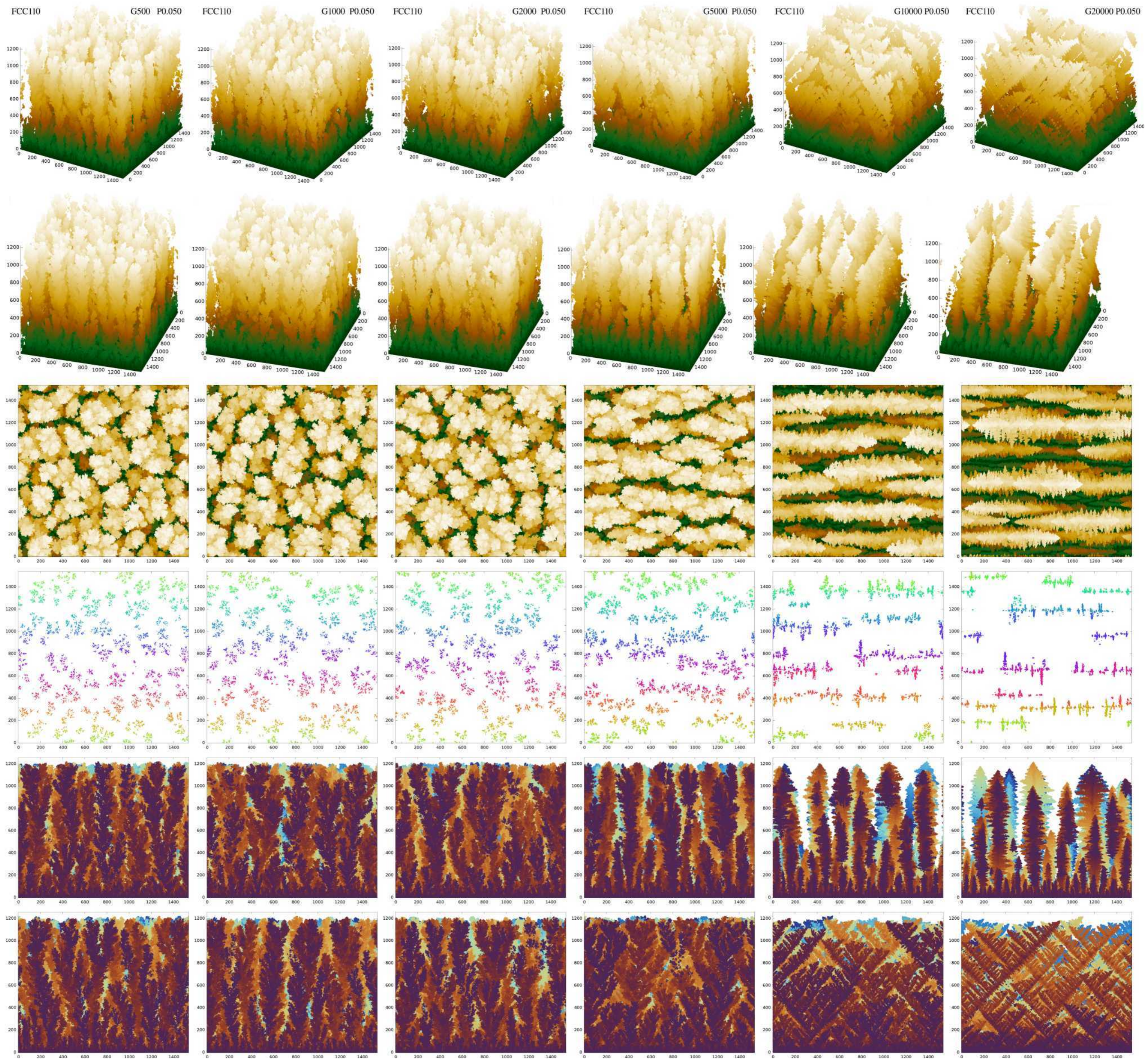

Figure S9: From top to bottom, perspective views, top views, cross sections at $\approx 2/3$ of the maximal height, and two lateral views of deposits grown on FCC(110) substrates with $P = 0.05$ and the indicated values of G.

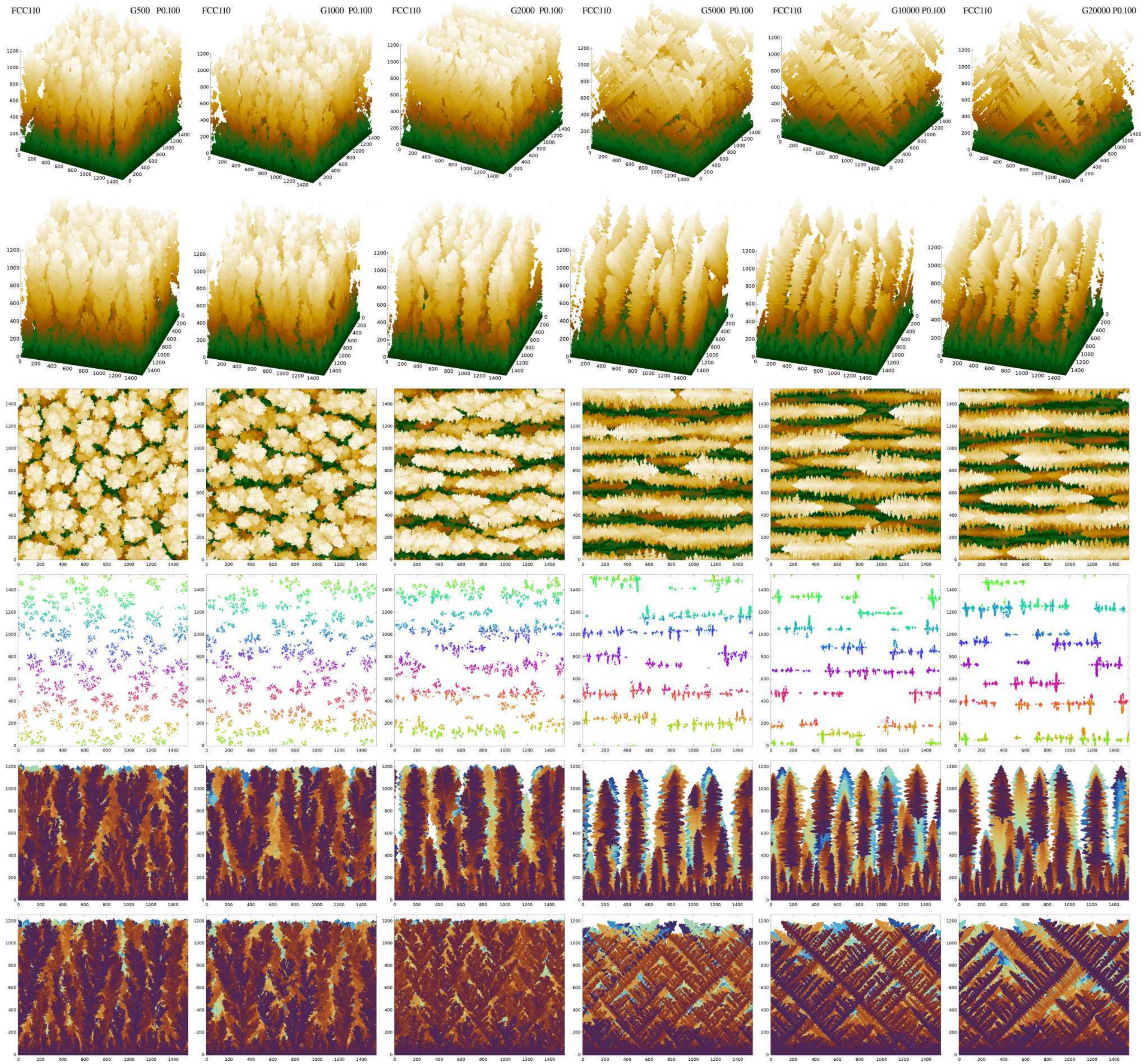

Figure S10: From top to bottom, perspective views, top views, cross sections at $\approx 2/3$ of the maximal height, and two lateral views of deposits grown on FCC(110) substrates with $P = 0.1$ and the indicated values of G.

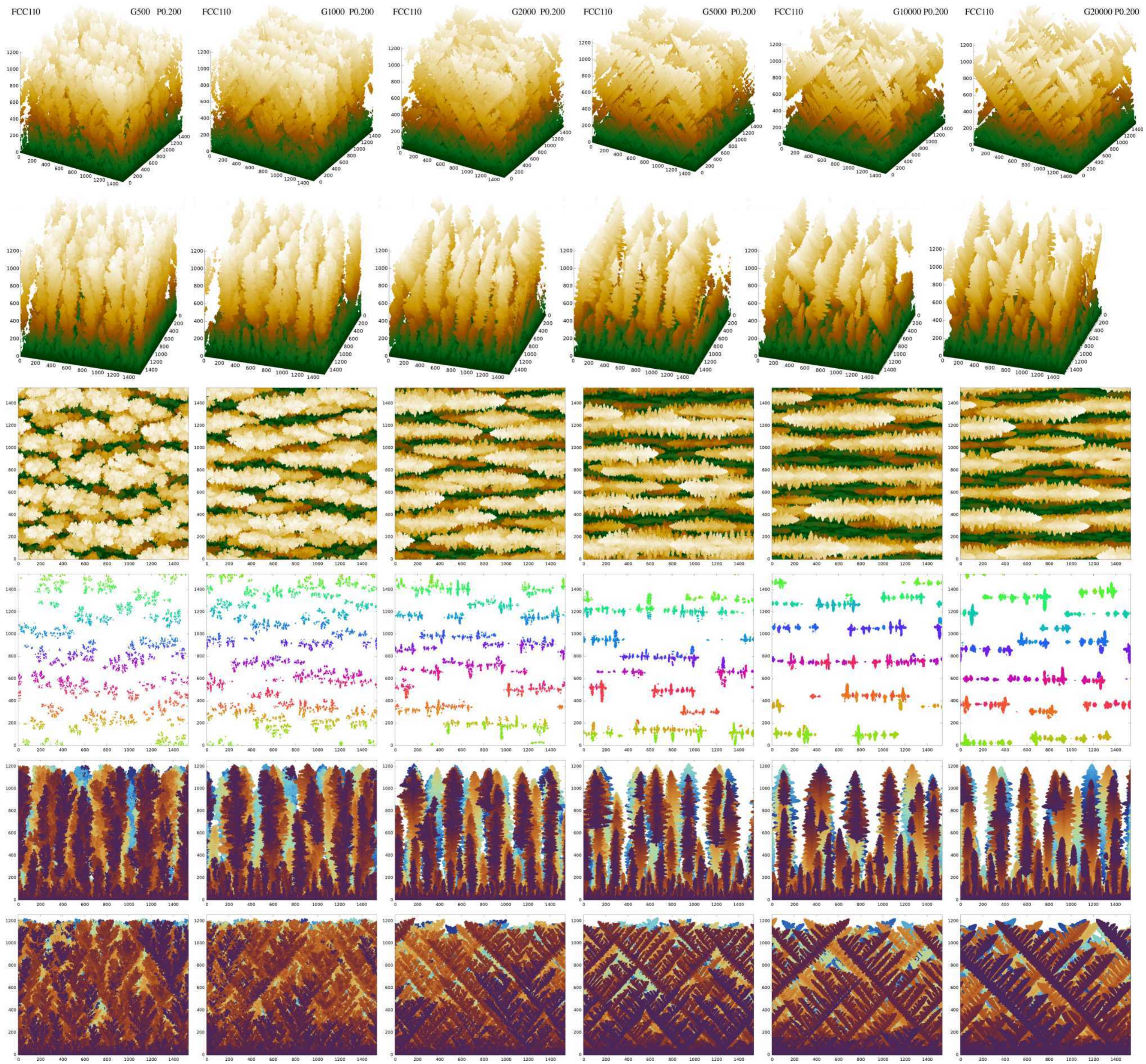

Figure S11: From top to bottom, perspective views, top views, cross sections at $\approx 2/3$ of the maximal height, and two lateral views of deposits grown on FCC(110) substrates with $P = 0.2$ and the indicated values of G.

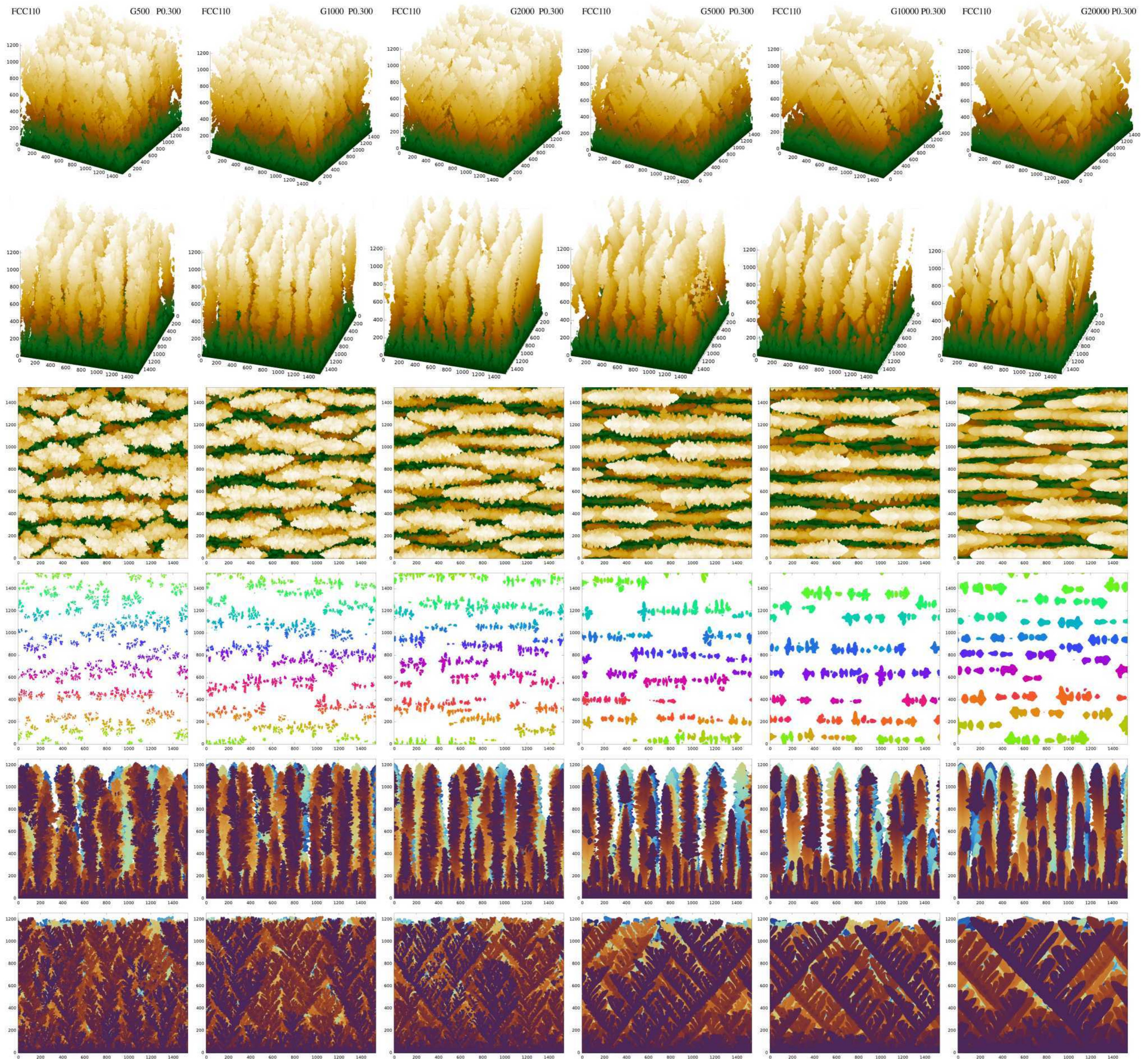

Figure S12: From top to bottom, perspective views, top views, cross sections at $\approx 2/3$ of the maximal height, and two lateral views of deposits grown on FCC(110) substrates with $P = 0.3$ and the indicated values of G.

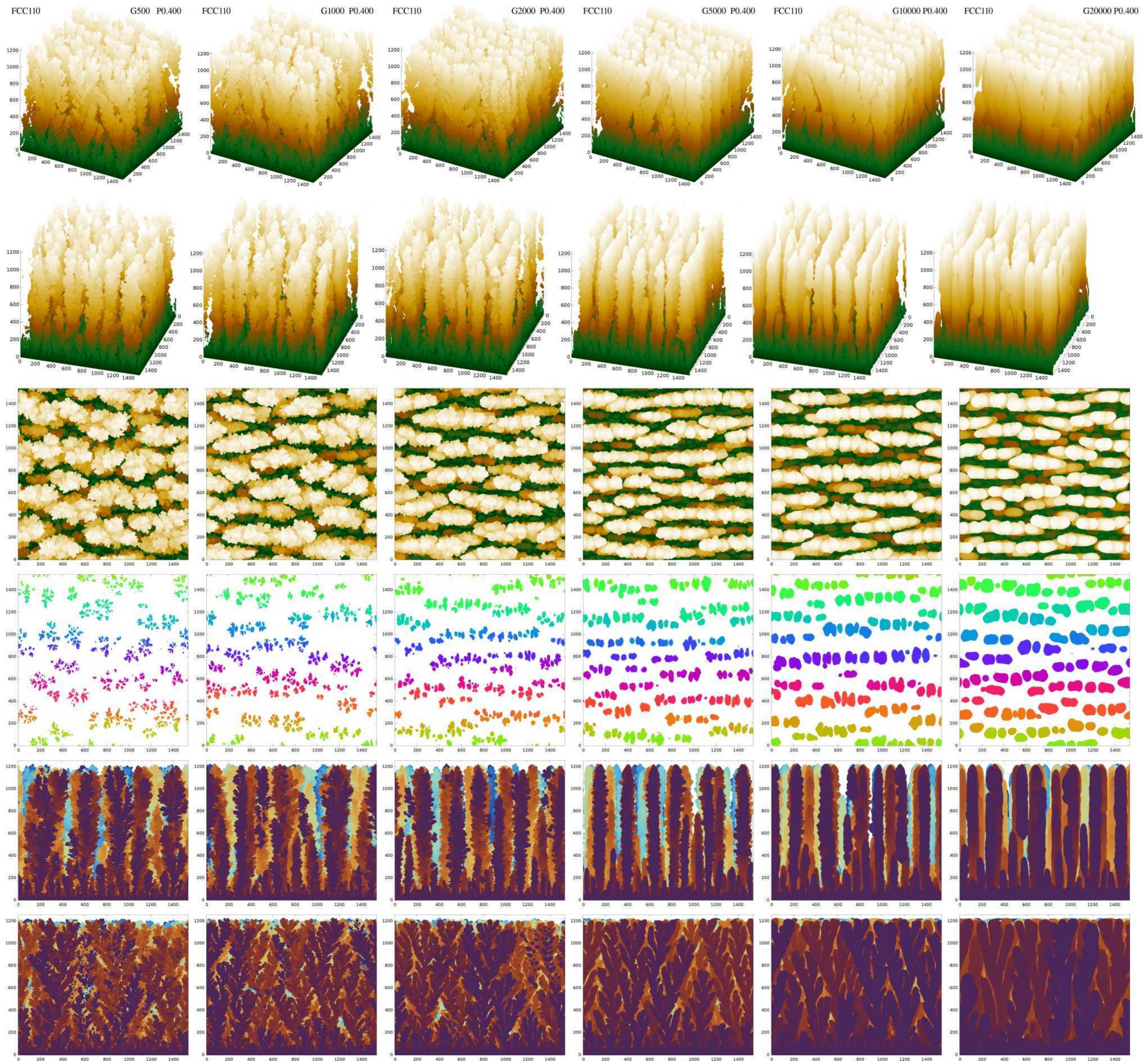

Figure S13: From top to bottom, perspective views, top views, cross sections at $\approx 2/3$ of the maximal height, and two lateral views of deposits grown on FCC(110) substrates with $P = 0.4$ and the indicated values of G.

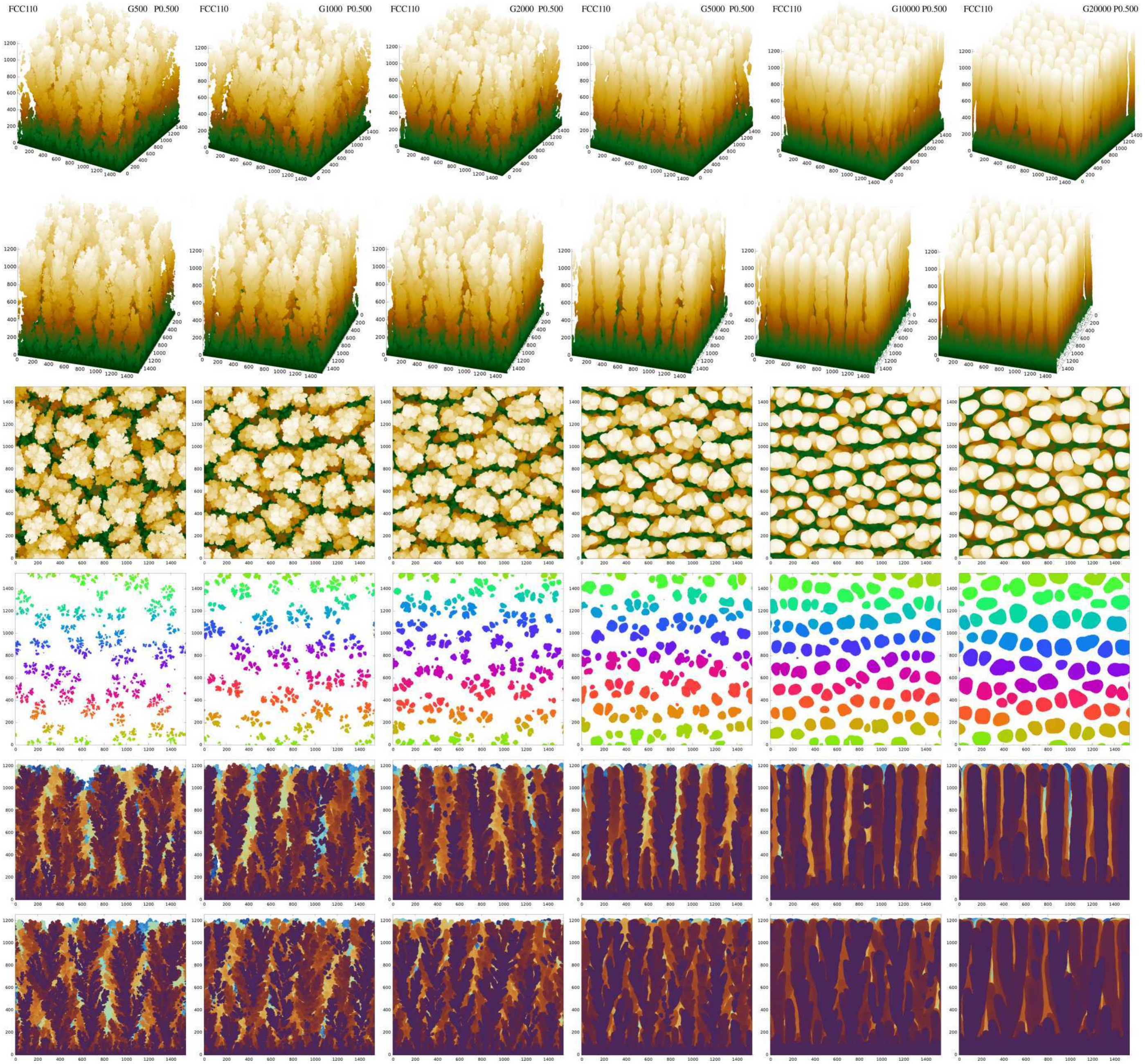

Figure S14: From top to bottom, perspective views, top views, cross sections at $\approx 2/3$ of the maximal height, and two lateral views of deposits grown on FCC(110) substrates with $P = 0.5$ and the indicated values of G.

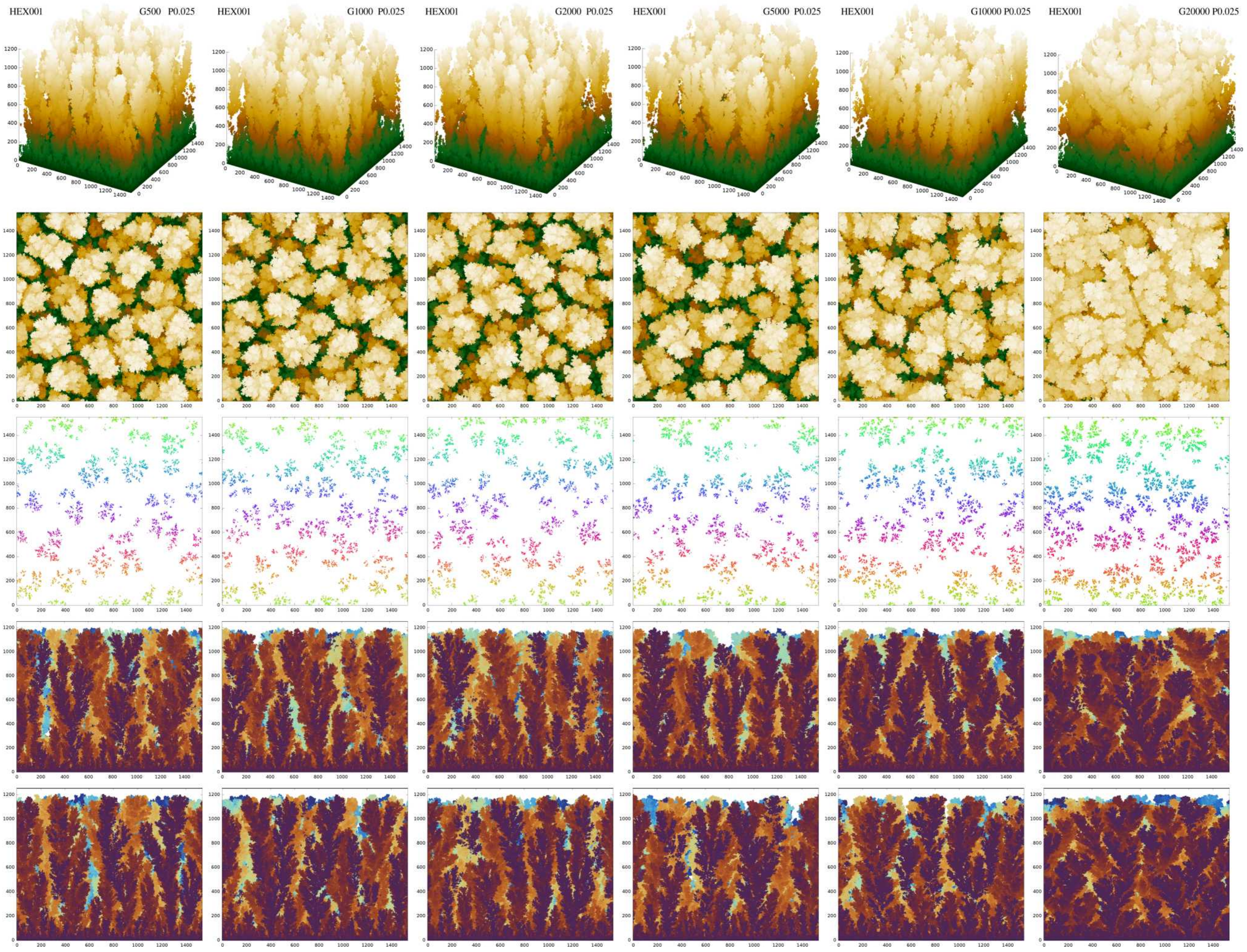

Figure S15: From top to bottom, perspective views, top views, cross sections at $\approx 2/3$ of the maximal height, and two lateral views of deposits grown on HCP(0001) substrates with $P = 0.025$ and the indicated values of G.

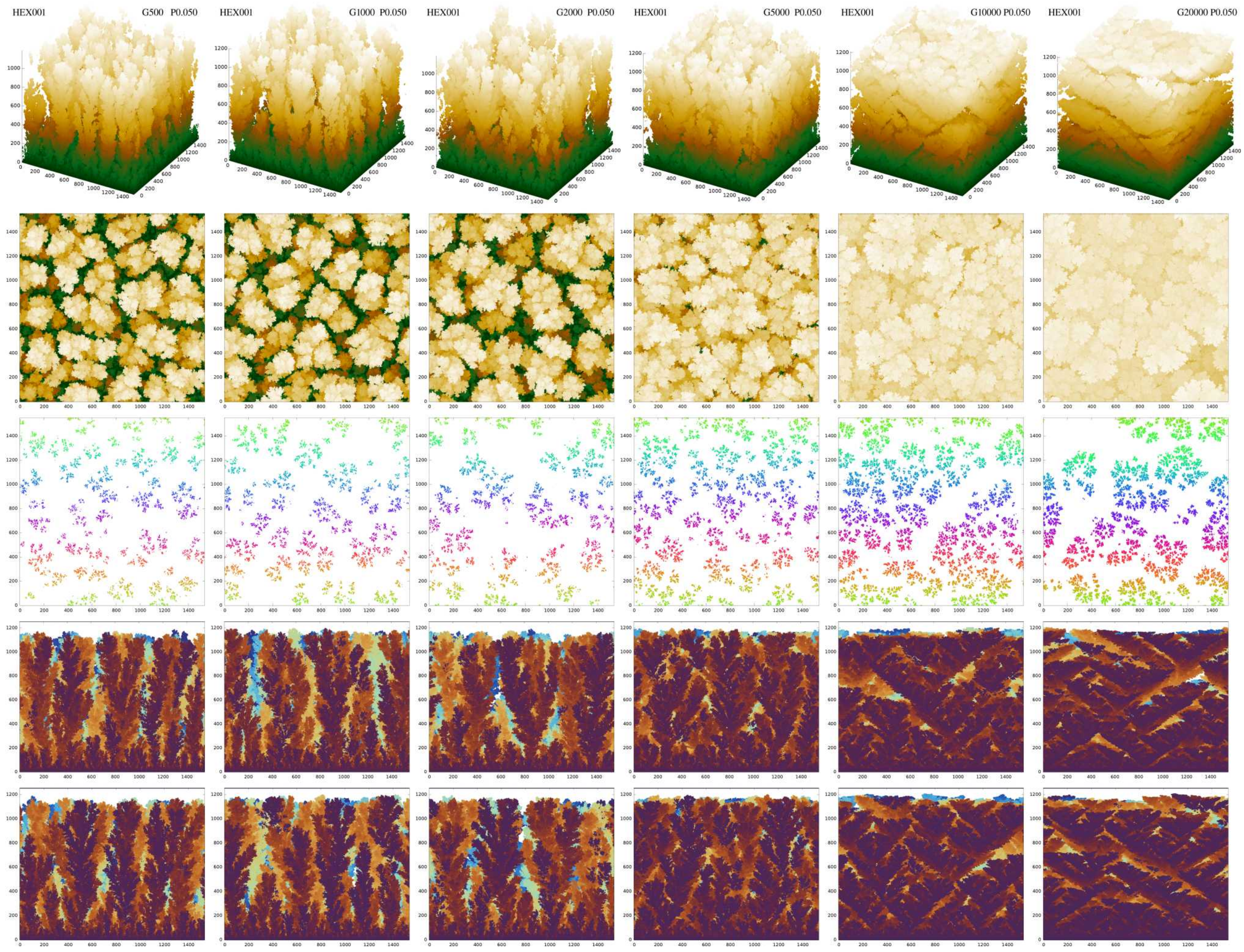

Figure S16: From top to bottom, perspective views, top views, cross sections at $\approx 2/3$ of the maximal height, and two lateral views of deposits grown on HCP(0001) substrates with $P = 0.05$ and the indicated values of G.

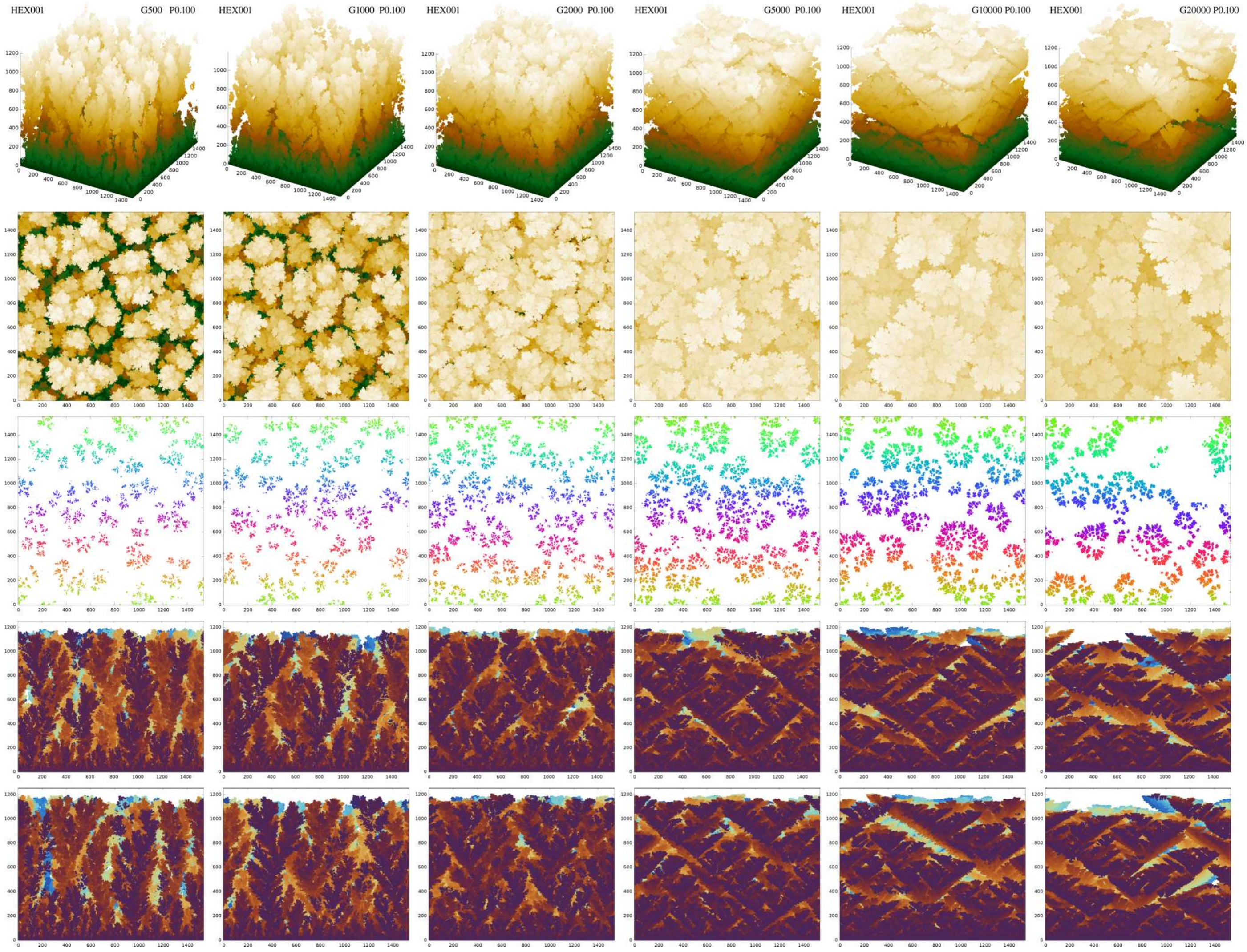

Figure S17: From top to bottom, perspective views, top views, cross sections at $\approx 2/3$ of the maximal height, and two lateral views of deposits grown on HCP(0001) substrates with $P = 0.1$ and the indicated values of G.

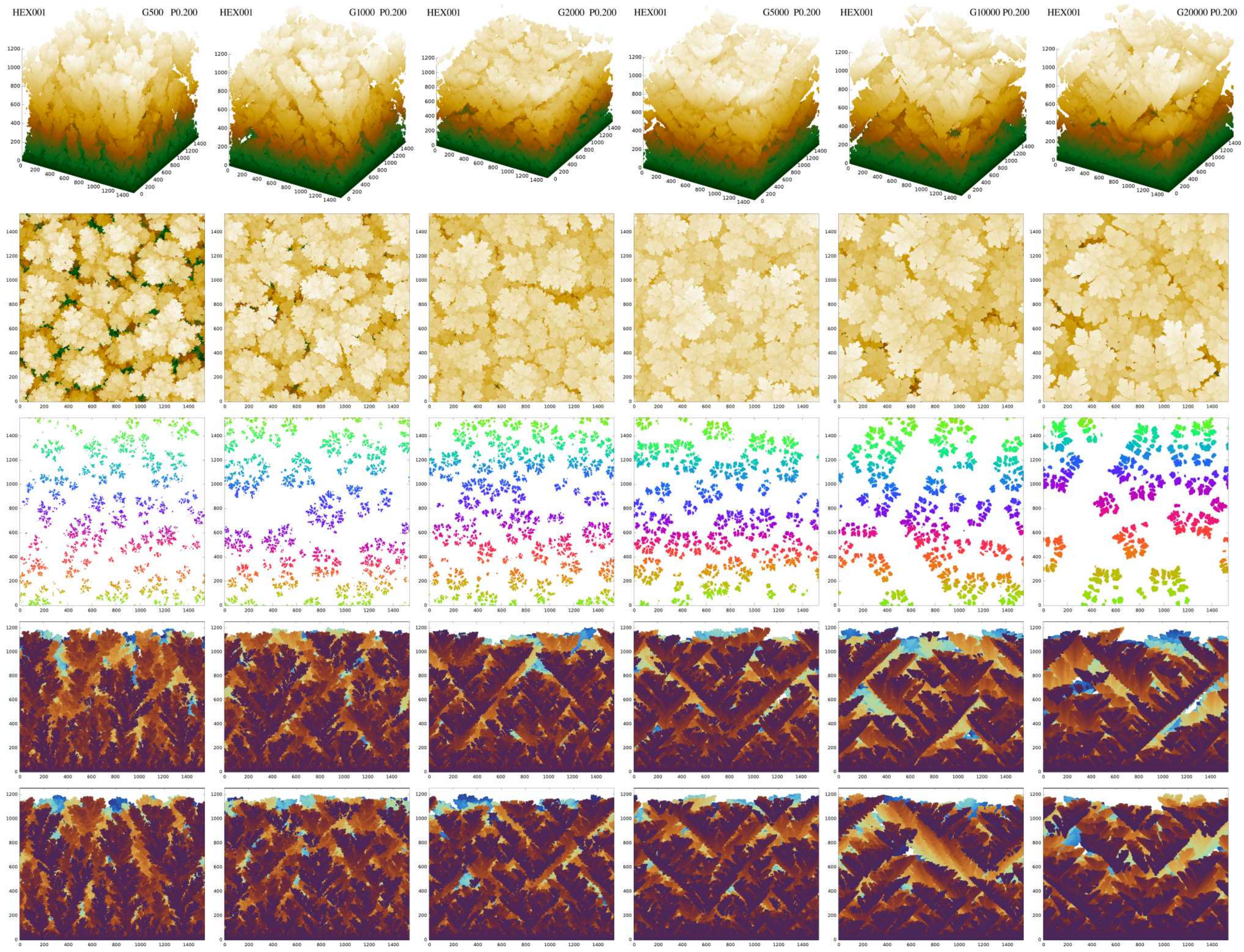

Figure S18: From top to bottom, perspective views, top views, cross sections at $\approx 2/3$ of the maximal height, and two lateral views of deposits grown on HCP(0001) substrates with $P = 0.2$ and the indicated values of G.

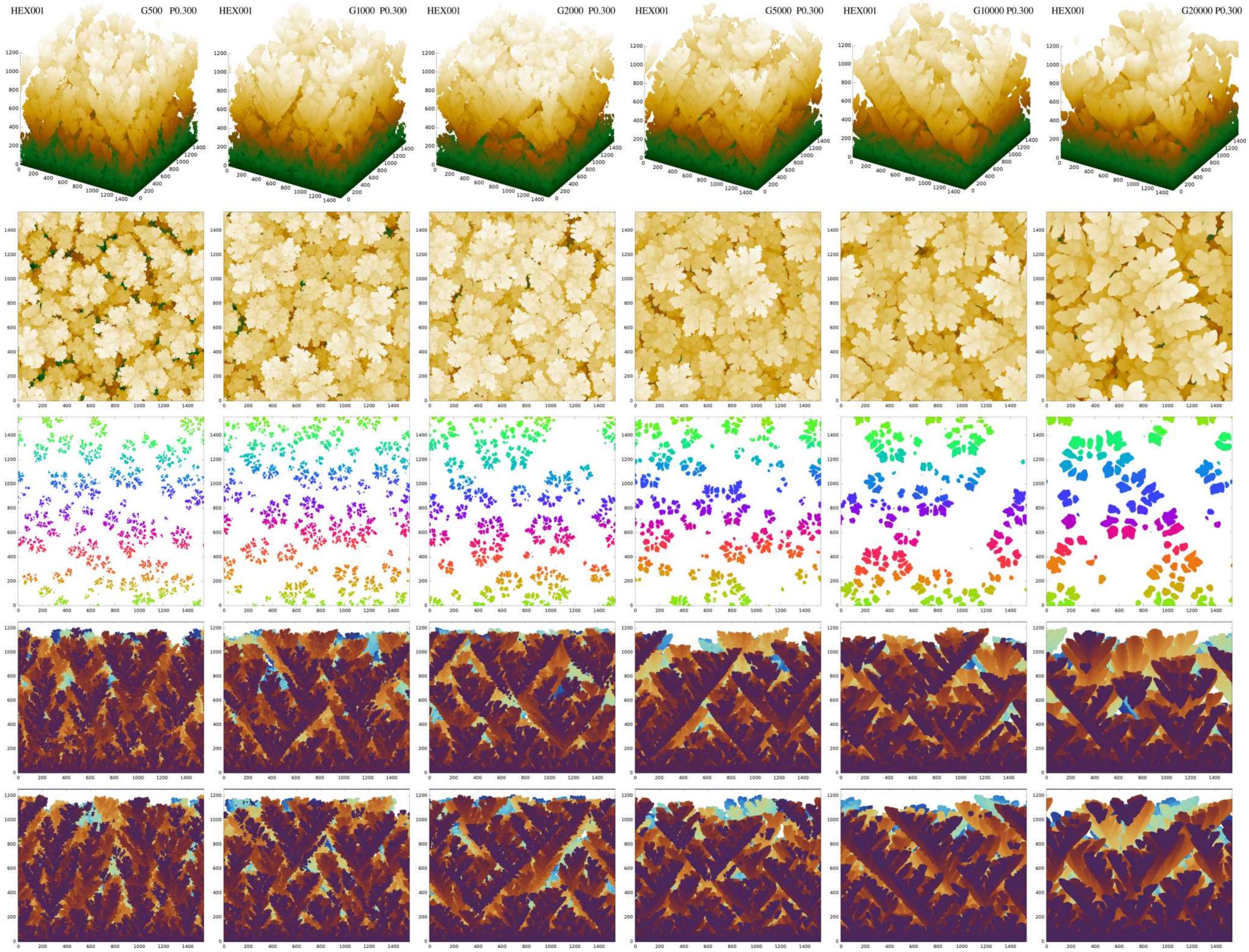

Figure S19: From top to bottom, perspective views, top views, cross sections at $\approx 2/3$ of the maximal height, and two lateral views of deposits grown on HCP(0001) substrates with $P = 0.3$ and the indicated values of G.

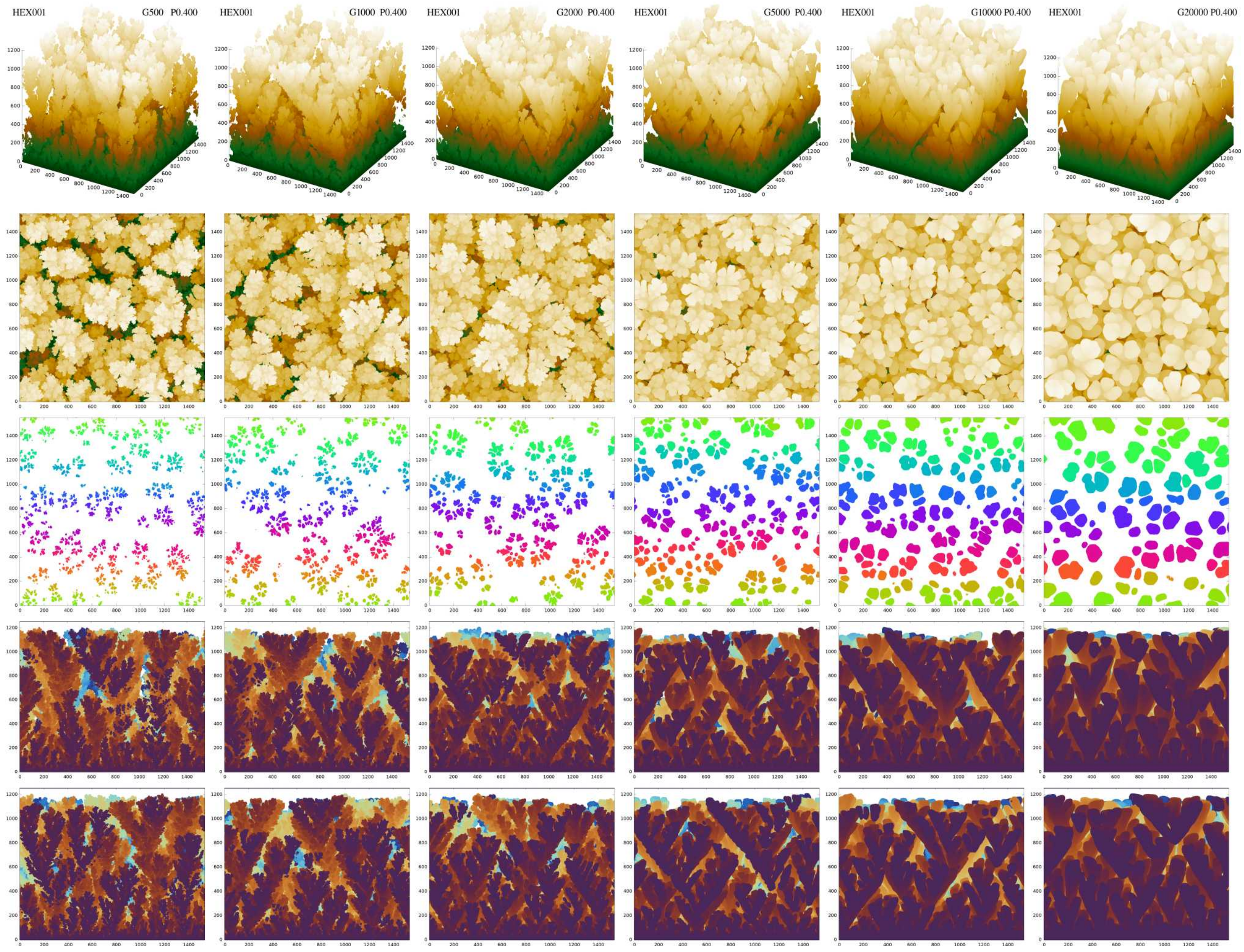

Figure S20: From top to bottom, perspective views, top views, cross sections at $\approx 2/3$ of the maximal height, and two lateral views of deposits grown on HCP(0001) substrates with $P = 0.4$ and the indicated values of G.

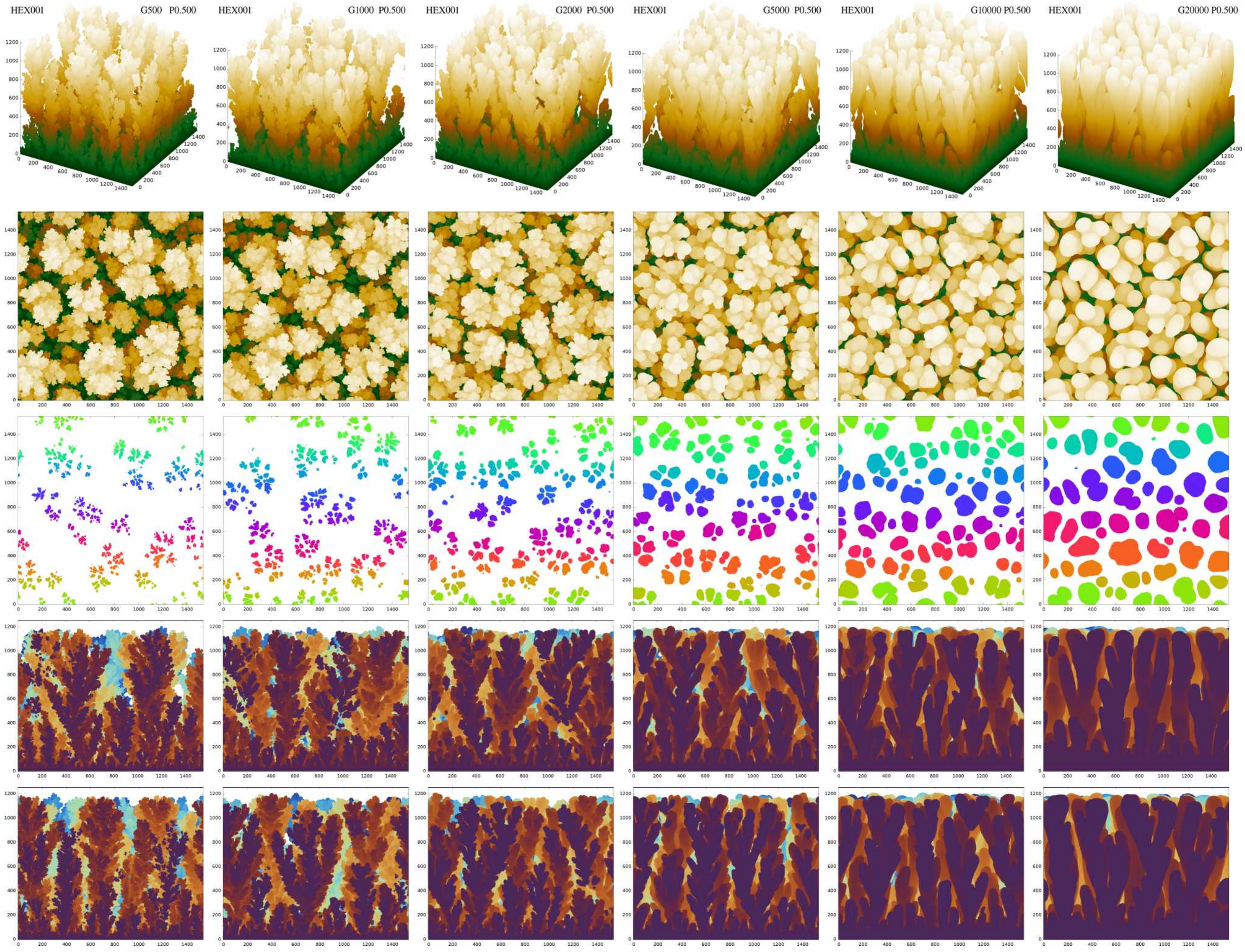

Figure S21: From top to bottom, perspective views, top views, cross sections at $\approx 2/3$ of the maximal height, and two lateral views of deposits grown on HCP(0001) substrates with $P = 0.5$ and the indicated values of G.
